# Quantifying the localized relationship between vector containment activities and dengue incidence in a real-world setting: A spatial and time series modelling analysis based on geo-located data from Pakistan

**DOI:** 10.1371/journal.pntd.0008273

**Published:** 2020-05-11

**Authors:** Nabeel Abdur Rehman, Henrik Salje, Moritz U G Kraemer, Lakshminarayanan Subramanian, Umar Saif, Rumi Chunara

**Affiliations:** 1 Computer Science and Engineering, Tandon School of Engineering, New York University, Brooklyn, New York, United States of America; 2 Institut Pasteur, Paris, France; 3 Department of Zoology, Oxford University, Oxford, United Kingdom; 4 Courant Institute of Mathematical Sciences, New York University, New York, New York, United States of America; 5 UNESCO Chair for ICTD, Lahore, Pakistan; 6 Department of Biostatistics, School of Global Public Health, New York University, New York, New York, United States of America; Chinese Center for Disease Control and Prevention, CHINA

## Abstract

Increasing urbanization is having a profound effect on infectious disease risk, posing significant challenges for governments to allocate limited resources for their optimal control at a sub-city scale. With recent advances in data collection practices, empirical evidence about the efficacy of highly localized containment and intervention activities, which can lead to optimal deployment of resources, is possible. However, there are several challenges in analyzing data from such real-world observational settings. Using data on 3.9 million instances of seven dengue vector containment activities collected between 2012 and 2017, here we develop and assess two frameworks for understanding how the generation of new dengue cases changes in space and time with respect to application of different types of containment activities. Accounting for the non-random deployment of each containment activity in relation to dengue cases and other types of containment activities, as well as deployment of activities in different epidemiological contexts, results from both frameworks reinforce existing knowledge about the efficacy of containment activities aimed at the adult phase of the mosquito lifecycle. Results show a 10% (95% CI: 1–19%) and 20% reduction (95% CI: 4–34%) reduction in probability of a case occurring in 50 meters and 30 days of cases which had Indoor Residual Spraying (IRS) and fogging performed in the immediate vicinity, respectively, compared to cases of similar epidemiological context and which had no containment in their vicinity. Simultaneously, limitations due to the real-world nature of activity deployment are used to guide recommendations for future deployment of resources during outbreaks as well as data collection practices. Conclusions from this study will enable more robust and comprehensive analyses of localized containment activities in resource-scarce urban settings and lead to improved allocation of resources of government in an outbreak setting.

## Author summary

Increasing global urbanization is having a profound effect on infectious disease risk, in particular presenting challenges for governments to allocate limited resources for controlling disease incidence at different locations within a city. With recent advances in the way data is collected, getting evidence about the efficacy of highly localized containment and intervention activities, which can lead to optimal deployment of resources, is possible. Here we develop methods for systematically analyzing the localized sub-city relationship between containment activities and disease incidence. Using precisely geo-located data on dengue containment activities and incidence, collected from two cities in Pakistan, we identify how the generation of new dengue cases changes in space and time with the application of seven types of containment activities. Our results show that activities targeted at adult stage of mosquito can reduce the generation of new dengue cases by 20% in their immediate vicinity and are effective up to a distance of 1km from the site of application. Further, given limitations due to the real-world nature of the data, we provide guidelines to improve data collection practices. The methods and results will enable governments and practitioners to improve the deployment of containment activities in an outbreak setting.

## Introduction

Infectious diseases pose significant public health and economic burden [1]. Dengue, one of most prevalent infectious diseases, is rapidly spreading with more than one half of the world’s population at risk for infection [2, 3]. The infection is a major public health problem in tropical and subtropical regions, where almost 400 million infections are estimated to occur each year [2]. Dengue virus is the most ubiquitous human arbovirus and is transmitted primarily by *Aedes aegypti* mosquitoes, a vector which also transmits several other global threats including Zika, chikungunya, and yellow fever [4]. Importantly, the virus disproportionately affects urban areas in developing countries; severe dengue is a leading cause of hospitalization and death among children and adults in urban areas in Asia, and Central and South America today [5]. This poses significant challenges for governments to allocate limited resources for the optimal control of disease at a sub-city scale [6, 7].

To date, the most common approaches to reducing the burden of dengue focus on containment of the vector population and broadly fall into three categories: (i) targeting vector breeding sites (source reduction); (ii) targeting vector at the larval stage; and (iii) targeting adult vector populations [8]. Despite the widespread use of containment activities, costing millions of dollars each year [9], the evidence base of their localized relationship with actual incidence is limited given the lack of reliable data collection methods, leading to less than optimal deployment of resources of governments.

Existing research in this domain has largely focused on controlled trials of individual activities [10–12], or use national and sub-national surveys to model the effect of containment at large spatial units [13]. Given the systematized nature of controlled trials, they generally focus on the effect of containment activities in a controlled environment; therefore the results may not be directly applicable to real-world settings, where external factors may impact the deployment and efficacy of containment activities [14]. Further, nearly all efforts to quantify the effect of activities on vector control use markers of vector presence (e.g., household/container indices, Breteau indices) as the main outcome of measure, and do not incorporate disease incidence directly [15]. However, the link between such vector measurements and dengue risk is poorly understood and a recent systematic review found little evidence of entomological indices such as the Breteau index being statistically associated with risks of dengue transmission [16, 17]. In addition to the above challenges, survey based studies, using aggregated counts over large spatial units, may often over-estimate or under-estimate the effect of a containment activity as such methods assume the instances of activities to be uniformly distributed across entire cities or states [18]. Given the localized effectiveness of a single containment activity instance, there is a need for analyses at similarly small (sub-city) spatial resolutions. Results of such analyses can also guide decision making, given the decisions that public health departments must make during outbreak situations. With recent advances in data collection practices such as mobile tools for automatic recording of digital data that can be linked to precise geo-locations [19], empirical evidence about the efficacy of highly localized containment and intervention activities, which can lead to optimal deployment of resources, is possible [20]. However, as containment activities are typically pragmatically deployed, they will not be random with respect to disease incidence, nor in relation to other types of containment activities, presenting challenges for analysis of such real-world observational data.

To address these challenges, we develop and assess two frameworks for understanding how the generation of new dengue cases changes in space and time with respect to application of different types of containment activities, using precisely geo-located data. This enables us to perform, to our knowledge, the first empirical study of the relationship of vector containment activities with actual dengue case incidence, at a local, sub-city resolution. Our study is made possible by a tremendously detailed and comprehensive system, the Punjab Anti-Dengue Activity Tracking System which was initiated as part of a massive effort to improve dengue surveillance in Pakistan, after the major dengue outbreak in 2011. As far as we are aware, this is the world’s largest dengue containment monitoring system, in which millions of containment activities since January 2012 have been recorded across two cities in Pakistan, each data point with precise geo-location. The data spans seven containment activities targeted at multiple stages of the life cycle of a mosquito. In order to improve the strength of conclusions based on this observational data we use two systematic statistical methods. Specifically, we aim to address biases based on the pragmatic nature of real-world containment activity deployment as well as other spatial and temporal factors as best as possible. The methods also generate results that can help guide decision makers, to assess both the reduction in probability of new cases immediately around cases which received each type of containment activity, and the relationship between the amount of each containment activity and a standardized epidemiological metric, the basic reproductive number, *R*_0_, of dengue. Based on results of our analysis, which showed limitations regarding for which activities robust conclusions could be drawn based on the nature of the activity deployment, we use this opportunity to conclude with feasible recommendations based on our findings from this real-world data analysis regarding how vector containment activities can be deployed more systematically during outbreak settings, to enable strong conclusions from real-world data.

## Materials and methods

### Data descriptions

**Fig 1:**
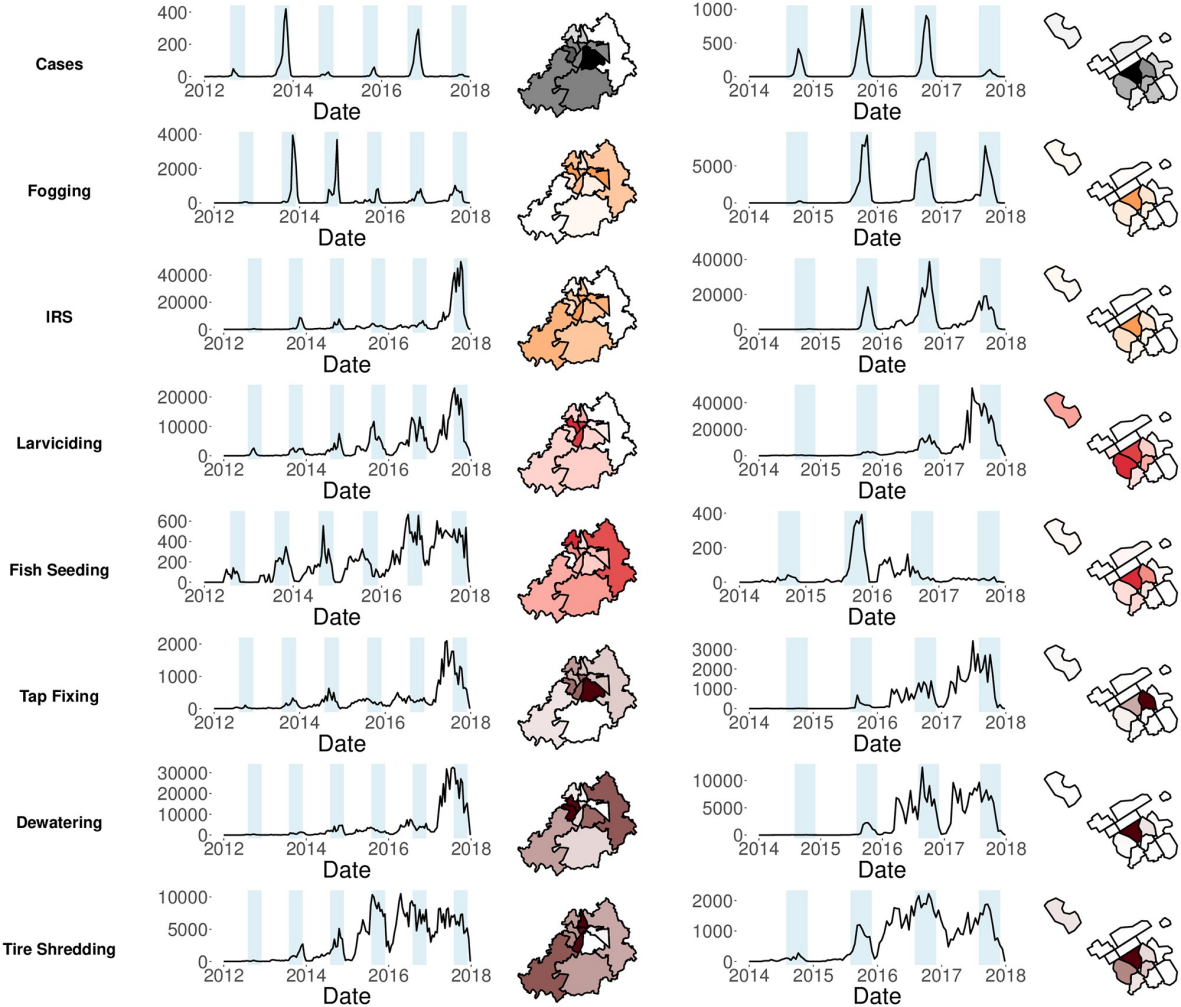
Spatial distribution and timeseries of dengue cases and containment activities. Spatial distribution and bi-weekly aggregate timeseries of dengue cases and containment activities between 2012 and 2017 in Lahore (left), and between 2014 and 2017 in Rawalpindi (right). Timeseries plots for each city show aggregated numbers from all sub-city spatial units. Spatial units colored to show relative distribution of cases and each containment activity within each city; lighter areas with lower numbers (total number of cases and containment activities summarized in Tables A and B in the appendix). Adulticides are color coded orange, larvicides coded red and source reduction activities coded as brown. Spatial units boundaries for each city are shown as black polygons. In Rawalpindi, spatial units are defined only in areas where cases occurred. All figures produced by the authors.

Data on containment activities were collected as part of an effort by the Punjab Information Technology Board (PITB) in Pakistan to improve accountability after the 2011 dengue outbreak in Pakistan [21]. Health care workers were equipped with smartphones and recorded pictures, before and after performing a containment activity using a mobile application, as verification that the activity had been performed (see Fig A in S1 Appendix for screenshot of smartphone application). These pictures, labelled with the type of activity, global positioning system (GPS) coordinates and the time-stamp of the activity were automatically submitted to a centralized server (see S1 Appendix section “Smartphone Application” for further details on data collection). We received the containment activities data for the cities of Lahore and Rawalpindi from PITB. The data consisted of 2,366,218 containment activity instances recorded in the city of Lahore between January 1, 2012 and December 31, 2017, and 1,610,941 recorded in the city of Rawalpindi between January 1, 2014 and December 31, 2017 (Fig 1). Seven types of containment activities are included: fogging spray and indoor residual spray (IRS) which are targeted at the adult mosquito population, larviciding and fish seeding which are targeted at the larval population, and tire shredding, dewatering, and tap fixing which are targeted at reducing breeding sites of mosquitoes (see S1 Appendix section “Containment Activities” for details on smartphone application used to collect data and description of individual activities; see Table A in S1 Appendix for tabulation of number of containment activities).

Data on confirmed dengue incidence from both cities was also received from PITB. Details of confirmed dengue incidence from all public hospitals in Punjab province of Pakistan are collected in a centralized server. Confirmation of dengue cases in hospitals in both cities is performed using enzyme-linked immunosorbent assay (ELISA) kits which measure anti-DENV IgM or IgG antibodies in patients’ serum [22]. Further details on the systematic methods used by hospitals can be found in the S1 Appendix section “Patient Portal and Criteria for Patients”. For our study we were provided access to a unique anonymous identifier for each dengue case along with the date of when they first experienced symptoms of dengue. Each patient’s home address is precisely tagged with geo-coordinates using smartphones; transformed linked coordinates were also made available for the study (spatial analysis), and spatially aggregate data for the time series analysis. See S1 Appendix section “Patient Portal and Criteria for Patients” for details on geo-tagging, transformation process to preserve privacy and laboratory confirmation details. Overall, the data consisted of 10,888 confirmed, geo-coded dengue cases reported in the cities of Rawalpindi (*n* = 7,890 between January 1, 2014 and December 31, 2017) and Lahore (*n* = 2,998 between January 1, 2012 and December 31, 2017) (Fig 1). As data on dengue serotype was not available, and the predominant serotype of dengue in Punjab was DENV-2 [23], we assumed all dengue patients were infected with the same serotype, consistent with previous research on dengue in Pakistan during similar time periods [24, 25].

City-wide daily mean temperature and mean precipitation estimates were obtained from the Pakistan Meteorological Department [26]. High-resolution population data was retrieved from WorldPop [27], and previously published work [28].

### Spatial signature modelling

#### Spatial dependence of cases

We developed a microscale spatial signature modeling approach to assess how the likelihood of presence of new dengue cases around existing cases (spatial dependence) changes with the deployment of each of the containment activities. To do so, we built on previous spatial signature models which assess spatial dependence of cases [29, 30]. Case clustering dynamics may be affected by exogenous heterogeneities that create spatial or temporal clustering (e.g. variation in population density, hospital and healthcare use, reporting rates, biases in where activities are deployed, population immunity, availability of vegetation and water for growth of vector in different locations and dengue seasonality) [29, 31]. Accordingly, to ensure results regarding containment activities in relation to cases are not biased by such factors (the “epidemiological context”) which would naturally lead to spatial clustering of cases, the first step was to simply identify the probability of observing new cases around existing cases within the two cities. To do so, we used a spatial dependence metric *τ*(*d*_1_, *d*_2_, *t*_1_, *t*_2_) which quantifies the likelihood of observing cases in the distance window *d*_1_ − *d*_2_ and temporal window *t*_1_ − *t*_2_ compared to if the clustering of cases was independent in space and time:

$$\begin{array}{c} \tau_{i}\left(d_{1},d_{2},t_{1},t_{2}\right)=\frac{Pr(\Omega_{i}\left(d_{1},d_{2},t_{1},t_{2}\right))}{Pr\left(\Omega_{i}\left(d_{1},d_{2},\cdot ,\cdot \right)\right)Pr\left(\Omega_{i}\left(\cdot ,\cdot ,t_{1},t_{2}\right)\right)} \end{array}$$ (1)

where Ω_*i*_(*d*_1_, *d*_2_, *t*_1_, *t*_2_) is the set of cases between *d*_1_ and *d*_2_ (in meters) and temporal window of *t*_1_ and *t*_2_ (in days) of case *i*; Ω_*i*_(⋅, ⋅, *t*_1_, *t*_2_) is the set of cases in temporal window *t*_1_ to *t*_2_ of case *i* independent of space, and Ω_*i*_(*d*_1_, *d*_2_, ⋅, ⋅) the set of cases within spatial window *d*_1_ and *d*_2_ of case *i*, independent of time. The overall spatial dependence of new cases appearing around cases labelled *s* (labelling is defined in the next subsection) is then estimated as:

$$\begin{array}{c} \begin{array}{cc} & {\hat{\tau }}_{s}\left(d_{1},d_{2},t_{1},t_{2}\right)=\\ & \frac{(\sum_{i=1}^{N}|\Omega_{i}\left(d_{1},d_{2},t_{1},t_{2}\right)|z_{i})\cdot (\sum_{i=1}^{N}|\Omega_{i}\left(\cdot ,\cdot ,\cdot ,\cdot \right)\left|z_{i}\right)}{(\sum_{i=1}^{N}|\Omega_{i}\left(d_{1},d_{2},\cdot ,\cdot \right)|z_{i})\cdot (\sum_{i=1}^{N}|\Omega_{i}\left(\cdot ,\cdot ,t_{1},t_{2}\right)\left|z_{i}\right)} \end{array} \end{array}$$ (2)

where *z*_*i*_ is 1 if the case is labelled *s*, *N* is the total number of cases in the dataset regardless of their label, and Ω_*i*_(⋅, ⋅, ⋅, ⋅) is the set of all cases in the dataset. For our analysis, we used a fixed time window of 30 days: *t*_1_ is selected as the day when the patient experienced first symptoms of dengue virus, and *t*_2_ = *t*_1_ + 30. This time window was chosen to ensure that cases considered were from the same transmission chain, though we performed a sensitivity analysis using additional time windows (see Fig C in S1 Appendix for sensitivity analysis using additional time windows). Dependence could then be observed across variation in the distance window. Understanding this baseline likelihood then allows us to then assess changes in the likelihood with the application of containment activities, while controlling for the epidemiological context.

#### Spatial signature of containment activities

To identify the impact of containment activities on the spatial dependence of dengue cases (the “spatial signature” of an activity) we first labelled all cases in the dataset as either a “containment” case, *s* = *a*, or a “non-containment” case, *s* = *b*, based on the presence of a containment activity in its spatio-temporal vicinity. A case was labelled as *s* = *a* if only the containment activity *a* was performed in a 20 meter radius and time window of the past 30 days of the case before the first symptom appeared. The short distance and time windows were chosen to ensure that the identified containment cases were concurrent with the most effective phase of the containment activity (see S1 Appendix subsection “Lagging of Containment Strategies” for details on the relationship between containment activities and adult mosquito population). Moreover, only cases for which a single containment activity was performed in the surrounding area were included in the analysis, to ensure only the effect of a single type of containment activity was being measured. A case was labelled a non-containment, *s* = *b*, if no containment activity was performed in a 20 meter radius and time window of the past 30 days of the case before the first symptom appeared.

To ensure results regarding containment activities in relation to cases are not biased by the epidemiological context, which could naturally lead to spatial clustering of cases, we match and make comparison between cases which have a similar epidemiological context. For a given set of containment cases labelled *a*, we selected a subset of cases, *a*′, such that each case in *a*′ has a matching non-containment case. A matching non-containment case was defined as a case which was within a radius of *m* meters, and was reported within 30 days of the containment case. For our analysis we present results for the value of *m* as 1,000, but also performed sensitivity analysis for values of 500 and 2,000 which did not change results. The choice of matching distance and time window were made to ensure that the matched non-containment cases were associated with similar latent variables, due to proximity in space and time to containment cases. As the efficacy of containment activities is generally in the immediate spatial vicinity, to confirm that the containment activity was not effective on the non-containment case at the matching distance, we tested sensitivity to this by including a minimum distance for the matched cases, which did not show any significant changes in the results (see Fig D in S1 appendix for results of constrained minimum distance range value).

For each containment case in *a*′, we randomly selected a matching non-containment case from the set of matching non-containment cases as *b*′_*a*_. The spatial signature of containment activity *a*, *ξ*_*a*_(*d*_1_, *d*_2_), was then calculated as the ratio of the spatial dependence of containment (${\hat{\tau }}_{a^{\prime }}$) to that of matching non-containment cases (${\hat{\tau }}_{{b^{\prime }}_{a}}$):

$$\begin{array}{c} \xi_{a}\left(d_{1},d_{2}\right)=\frac{{\hat{\tau }}_{a^{\prime }}\left(d_{1},d_{2}\right)}{{\hat{\tau }}_{{b^{\prime }}_{a}}\left(d_{1},d_{2}\right)} \end{array}$$ (3)

We performed 100 bootstrapping iterations, in each iteration randomly selecting a matching non-containment case for each containment case, and used these to present confidence intervals for the results. Values of *ξ*_*a*_(*d*_1_, *d*_2_) below 1 signify that the relative probability of a new case appearing in distance window (*d*_1_ to *d*_2_), around a case which was in proximity of a containment activity, *a*, is lower compared to that of a matching non-containment case, after adjusting for underlying clustering in space and time. Thus a value of *ξ*_*a*_(*d*_1_, *d*_2_) below 1 would be consistent with a positive impact from the containment activity. Values of *ξ*_*a*_(*d*_1_, *d*_2_) around 1 indicate no impact of the activity.

This method is useful for decision making during an outbreak, as it enables an understanding of not only how each containment activity was associated with a reduced probability of new cases in the immediate time and location vicinity of an existing case, but also the maximum distance up to which that probability was reduced.

### Time-series modelling

To understand the relationship of containment activities with transmission potential of the outbreak, we modelled dengue transmission based on our Pakistan case data, augmenting a time-series suspected infected recovered model (TSIR) of viral incidence to include the effect of containment activities as part of the transmission rate. TSIR models have previously been used to reconstruct dengue dynamics in Asia [24, 32]. Such modeling frameworks account for the (e.g non-linear) processes underlying disease dynamics and can be used to account for the partial-nature of the observed data (e.g. only the number of new infections at a given time point). As well, this model enables assessment of the transmission potential through a standardized metric, *R*_0_, the reproductive number of dengue. The reproductive number can be defined as the number of secondary infections a primary infection can cause over the course of its infectious period [33]. If *R*_0_ is greater than 1, then the disease will spread exponentially, while an *R*_0_ below 1 means that the disease will not spread.

Unlike the spatial dependence analysis, this methodology also allows us to incorporate compound effects of multiple containment activities. Though, in contrast to the spatial dependence analysis, the basic TSIR model does not account for varying epidemiological contexts across locations (such latent factors are assumed to be the same in each location considered). To limit potential variability in unmeasured factors that could affect transmission, we restrict study of the relationship between containment activities and dengue incidence to each city separately, and we used sub-city size spatial units at which to perform the analysis. Accordingly, incidence and containment data were mapped to sub-city spatial units (Fig 1 and see S1 Appendix subsection “Spatial Unit Definition” for details on sub-city partitions). Finally, we accounted for environmental drivers such as rainfall and temperature, to account for variation in vector population density across seasons and years, and population density to account for proximity of individuals to each other increasing the likelihood of transmitting disease [34]. Transmission of dengue within each of these spatial units was then modelled at a time step of two weeks (bi-weekly), which roughly coincides with the generation time of dengue, and has been used in previous dengue TSIR models [24, 35].

The number of cases in each spatial unit was first reconstructed to account for under-reporting. To do so, the reported number of cases at each time step, $I_{i}^{\left(r\right)}\left(t\right)$, are first smoothed, $I_{i}^{\left(s\right)}\left(t\right)$, then multiplied with the inverse of the reporting rate *rr*. This product, at each time step, is used as the mean of Poisson distribution:

$$\begin{array}{c} I_{i}\left(t\right)∼Poisson\left(\frac{I_{i}^{\left(s\right)}\left(t\right)}{rr}\right) \end{array}$$ (4)

where *I*_*i*_(*t*) is the actual infected number of cases in time *t*, in spatial unit *i*, computed from the Poisson distribution and dependent on the number of reported cases during the same time step in the spatial unit (see Tables F and Table G in S1 Appendix for aggregated reported cases, aggregated reconstructed cases, area and population for spatial unit in Lahore and Rawalpindi). This reconstruction methodology, used in previous infectious disease modeling work [36], gives the advantage of capturing tails of the epidemic curve in a realistic and continuous manner. We used a value of *rr* = 0.265 based on a survey published by the Pakistan Bureau of Statistics [37]. Given the large number of public and private hospitals in both cities, we found it fair to assume that reporting rate was constant across all spatial units. Further discussion on reporting rate and health care system in the cities can be found in the S1 Appendix section “Background of Study Setting and Disease Reporting”.

The general TSIR model, for each spatial unit, *i*, was then defined via the following equations:

$$\begin{array}{c} I_{i}\left(t+1\right)=\beta_{i}\left(t\right)\frac{S_{i}\left(t\right)}{N_{i}\left(t\right)}I_{i}^{\alpha_{i}}\left(t\right)\epsilon \end{array}$$ (5)

$$\begin{array}{c} S_{i}\left(t+1\right)=S_{i}\left(t\right)-I_{i}\left(t+1\right)+\rho N_{i}\left(t\right)-\phi S_{i}\left(t\right) \end{array}$$ (6)

$$\begin{array}{c} R_{i}\left(t+1\right)=R_{i}\left(t\right)+I_{i}\left(t\right)-\phi R_{i}\left(t\right) \end{array}$$ (7)

where *t* is recorded at an interval of 2-weeks (bi-weekly) as discussed above [24, 35]. *I*_*i*_(*t*), *S*_*i*_(*t*), *R*_*i*_(*t*) and *N*_*i*_(*t*) are the infected, susceptible, recovered and total population during time step *t* in spatial unit *i*, *ρ* is the bi-weekly birth rate, *ϕ* is the bi-weekly death rate, *α*_*i*_ is the mixing parameter in spatial unit *i*, and *β*_*i*_(*t*) is the transmission rate during time step *t*. The error term *ϵ* is assumed to be an independent and identically log-normally distributed random variable.

Bi-weekly environmental parameters values, available at this resolution for the entire study period, were incorporated in the transmission rate. Population density was also incorporated into the transmission rate. Containment activities were also modelled as part of the transmission rate. This is due to the fact that containment activities reduce the contact rate between humans and mosquitoes, which results in a reduction of the transmission rates from human to mosquito to human [38]. Containment activities can leave residue making them effective not just during the week they were applied but also in subsequent weeks. Larviciding activity prevents the growth of mosquito larvae for up to 45 days [39]. Tire shredding and chemical treatment of tires prevents the growth of larvae for up to 28 days [39]. Indoor Residual Spray (IRS) sticks on walls for up to 90 days [40]. In contrast, fogging spray disseminates in the air and generally does not leave a residue [41]. Tilapia fish, which are used in the fish seeding activity have a life expectancy of approximately 9 years [42]. These residues were thus accounted for in the modelling approach by assuming that the activities are effective in subsequent weeks of application, and their amount decays exponentially for the durations mentioned above. Hence during a given time-step, the total number of containment activities in a spatial unit was assumed to be the number of activities applied during the time-step and the residues from previous weeks. It should be noted that these residues are measured under controlled laboratory conditions, and could vary depending conditions in a real world setting– though there is no straightforward way for us to quantify this variation.

Accordingly, to incorporate containment activities into the transmission parameter, Eq 5 was rearranged as follows:

$$\begin{array}{c} log\left(\beta_{i}\left(t\right)\right)+\alpha_{i}*log\left(I_{i}\left(t\right)\right)=log\left(I_{i}\left(t+1\right)\right)+log\left(N_{i}\left(t\right)\right)-log\left(S_{i}\left(t\right)\right)-\epsilon \end{array}$$ (8)

and the transmission rate, as described above, was substituted with:

$$\begin{array}{c} log\left(\beta_{i}\left(t\right)\right)=\sum_{a}\theta_{a}C_{i,a}\left(t-l_{a}\right)+\sum_{j}\theta_{j}E_{j}\left(t-l_{j}\right)+\theta_{p}D_{i}\left(t\right) \end{array}$$ (9)

where *l*_*a*_ and *l*_*j*_ are the delays in time steps which were added to containment activities *a* and environmental parameters *j* respectively. *C*_*i*,*a*_(*t* − *l*_*a*_) is the number of times containment activity *a* was performed in spatial unit *i* during the time step (*t* − *l*_*a*_). *E*_*j*_(*t* − *l*_*j*_) is the value of environmental parameter *j* during time step (*t* − *l*_*j*_). *D*_*i*_(*t*) is the population density in spatial unit *i* computed by dividing the population of the spatial unit with the area of the spatial unit. *θ*_*a*_, *θ*_*j*_ and *θ*_*p*_ are the parameters, to be estimated, which relate containment activities, environmental parameters and population density to the transmission rate *β*. Delays in dengue transmission between human and vector were accounted in the model for realistic modelling of the transmission of dengue and effect of containment activities over time. Details on delays to account for vector life cycle, transmission and containment activity residues are described in S1 Appendix subsection “Vector Life Cycle and Delayed Effect of Containment Activities”.

A shape constrained additive model (SCAM) was used to fit the relationship in Eq 8 and identify the parameters *θ*_*a*_, *θ*_*j*_, *θ*_*p*_ and *α*_*i*_, that give the best fit across all time points *t*, and all spatial units *i*. This allowed us to model the transmission parameter of dengue, *β* as a time-varying function of containment activities, environmental parameters and population density. Shape constrained additive models are an extension of generalized additive models (GAMs) which provide the advantage of using existing knowledge about the relationship of the response variable with the explanatory variables [43, 44]. This prevents noise from being included in the shape of splines from the GAM; a similar approach has been used previously to fit the transmission rate parameter for dengue and Zika [45, 46]. Containment activities were fit as monotonically decreasing splines while environmental parameters and population density were fit as monotonically increasing splines. The mixing parameter was fitted as a linear coefficient. The model was optimized using maximum likelihood and we used the “SCAM” package in R to fit the model.

Using the estimates of *θ*_*a*_, *θ*_*j*_, and *θ*_*p*_ we identified the variation in *R*_0_ (reproductive number of dengue) by variation in the amount of each containment activity. The *R*_0_ is calculated using the following equation:

$$\begin{array}{c} R_{0_{i}}\left(t\right)=\frac{\beta_{i}\left(t\right)}{\gamma } \end{array}$$ (10)

where, γ is the recovery rate and is equal to 1 time step in our study, given the fact that infected patients are immediately admitted in the hospital and removed from the infected population [24].

To assess the utility of containment data in modelling of dengue transmission, we trained additional variants of the TSIR model using only environmental parameters and population density. Additional details on initial conditions, and sensitivity analyses of parameters used in the study are discussed in the S1 Appendix subsection “Model Parameters, Initial Conditions and Fit”.

The TSIR modeling approach quantifies the relationship of containment activity deployment with number of dengue cases using a standard epidemiological parameter, the reproductive number. However, unlike the spatial signature analysis, this modelling approach does not account for heterogeneities in latent spatial attributes such as different clustering patterns of dengue cases in different locations. Hence, we performed the time series analysis separately for each city. Moreover, we used as small a spatial unit as possible; each city is divided into sub-city spatial units based on administrative boundaries and clusters of dengue cases (*n* = 10 for Lahore and *n* = 14 for Rawalpindi; see S1 Appendix subsection “Spatial Unit Definition” for details on sub-city partitions) to decrease latent heterogeneities, and transmission of dengue is modelled with respect to these sub-city units.

### Ethics

The data was collected as part of public health practice, and secondary use of the data here did not include any identifying information. No personally identifiable information of patients of any kind was exposed to us during the study. Details on steps to ensure no identifying information was accessed are described in detail in the “Patient Portal and Criteria for Patients” section (appendix) and “Time-series modelling” section.

## Results

Fig 1 illustrates the spatial and temporal distribution of dengue cases and containment activities across both cities. In both Lahore and Rawalpindi, we observed high dengue activity during the post monsoon months, September–November. In addition we observed that activities targeted at the adult stage of mosquitoes (IRS and fogging) were predominantly performed during the high dengue activity season. In contrast, activities targeted at the larval stage and breeding sites of mosquitoes were performed throughout the year, but the amount of containment activities was increased during the dengue season due to higher risk of infection. The spatial distribution of data showed that activities targeted at the adult stage of mosquitoes are limited to areas where dengue cases appear. Targeting of other containment activities was more spread out but were mostly targeted in areas where dengue cases appeared. Indeed, we can see how containment activities were administered in a pragmatic nature, and analyses needed to account for this.

### Spatial signature of containment activity instances

**Fig 2:**
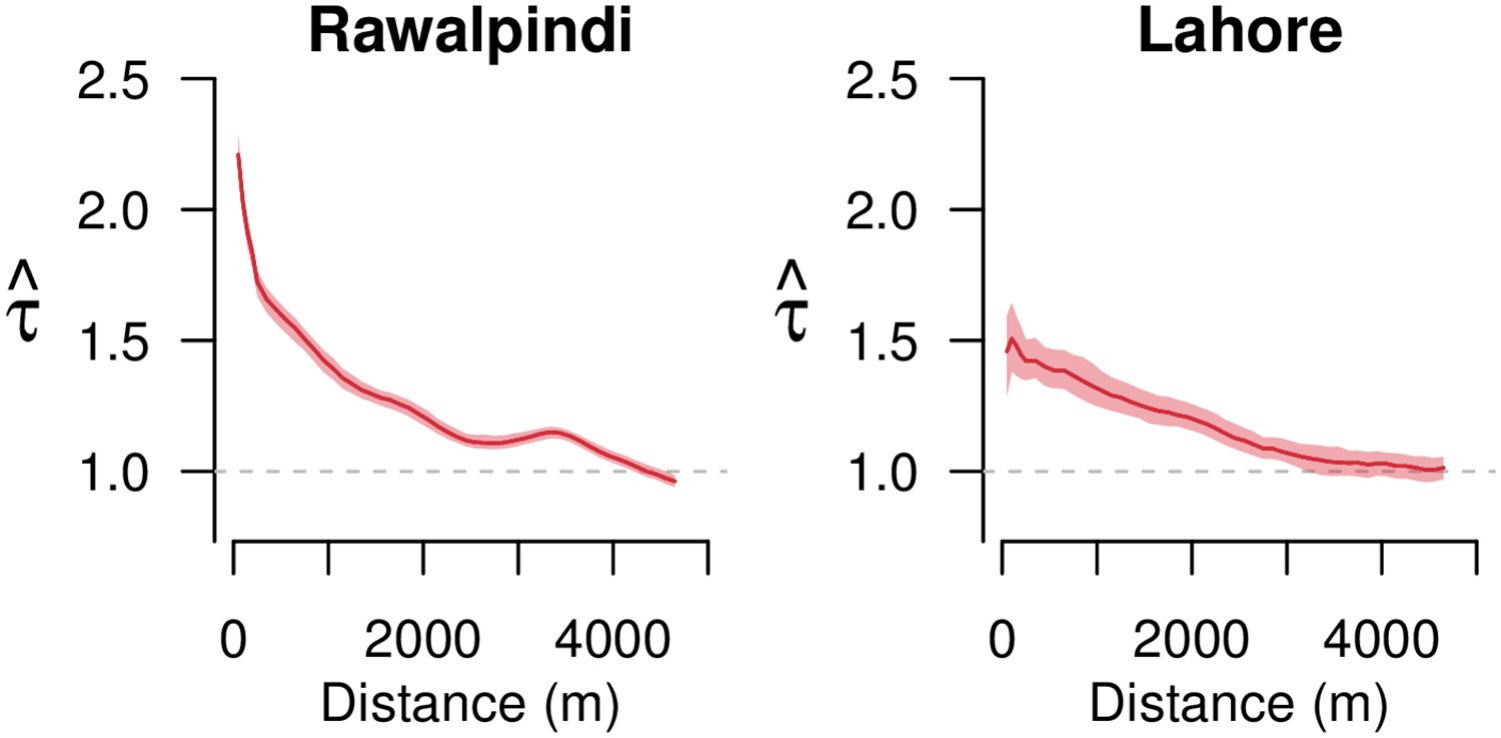
Spatial dependence of cases in Rawalpindi and Lahore. Spatial dependence of cases occurring within 30 days of index cases in Rawalpindi and Lahore. The spatial window of the analysis (*d*_2_ minus *d*_1_) is maintained at 500 m when *d*_2_ is greater than 500 m, and observations are made by sliding the window at intervals of 100 m. For *d*_2_ less than 500 m, *d*_1_ is equal to zero and observations are made by increasing *d*_2_ at intervals of 100 m. Spatial dependence estimates are plotted at midpoint of the spatial window. The time window *t*_2_ − *t*_1_ is set to 30 days. 95% CI is shown as shaded area around estimate.

Examining the overall spatial dependence between dengue cases in Rawalpindi and Lahore, we observed that closer to existing cases there was a higher probability of observing new cases, relative to if the clustering of dengue cases was independent in space and time (Fig 2). As the distance from existing cases increased, this increased probability decreased, until the value reached 1, where the probability of observing new cases was similar to if the clustering of the cases was uniform in space and time. This result, indicating increased likelihood of observing new cases closer to existing cases, indicated that there was a spatial dependence that may demonstrate the efficacy of containment activities, but that we needed to control for this baseline clustering before doing so.

Specifically, we observed a 2.21 times (95% CI 2.14–2.28) and 1.46 times (95% CI 1.29–1.59) increased probability, in Rawalpindi and Lahore respectively, of observing a case between *d*_1_ = 0 m and *d*_2_ = 100 m radius and within 30 days (*t*_2_—*t*_1_; where *t*_1_ is the day when the index case developed first symptoms) of an index case, relative to the probability of a case occurring if clustering was independent in space and time. These probabilities fall to 1.33 (95% CI 1.29–1.36) and 1.28 (95% CI 1.22–1.35) at a distance range *d*_1_ = 1,000 m and *d*_2_ = 1,500 m, and 1.0 (95% CI 0.98–1.03) and 1.0 (95% CI 0.97–1.06) at a distance range *d*_1_ = 4,100 m and *d*_2_ = 4,600 m, in Rawalpindi and Lahore respectively (Fig 2). As evident from the results, at closer distance range the spatial dependence of dengue cases in Lahore was lower compared to Rawalpindi. The variation in spatial dependence across different locations can be attributed to a multitude of factors including baseline immunity and variation is external factors such as landscape features, which provided breading locations for mosquitoes. Thus, this variation in spatial dependence of cases, across different locations and times, should be explicitly accounted for when studying the relationship between containment activities and reduction in probability of new cases.

Next, we studied the result of different containment activities on the spatial dependence between cases. To ensure that we are not studying the compound effect of different containment activities together, we identified cases which were in spatio-temporal vicinity of a single containment activity instance. In addition, given the variation in spatial dependence of cases, we only included those cases in our study which had similar epidemiological context. Of the 9,268 geo-tagged cases in Rawalpindi and Lahore, between 2014 and 2017, 531 were labelled with IRS (had this containment activity performed in the immediate spatio-temporal proximity), followed by larviciding (*n* = 275) and fogging (*n* = 162) (see Table B in S1 Appendix for breakdown of the number cases and number of cases with a matching control across containment activities).

**Fig 3:**
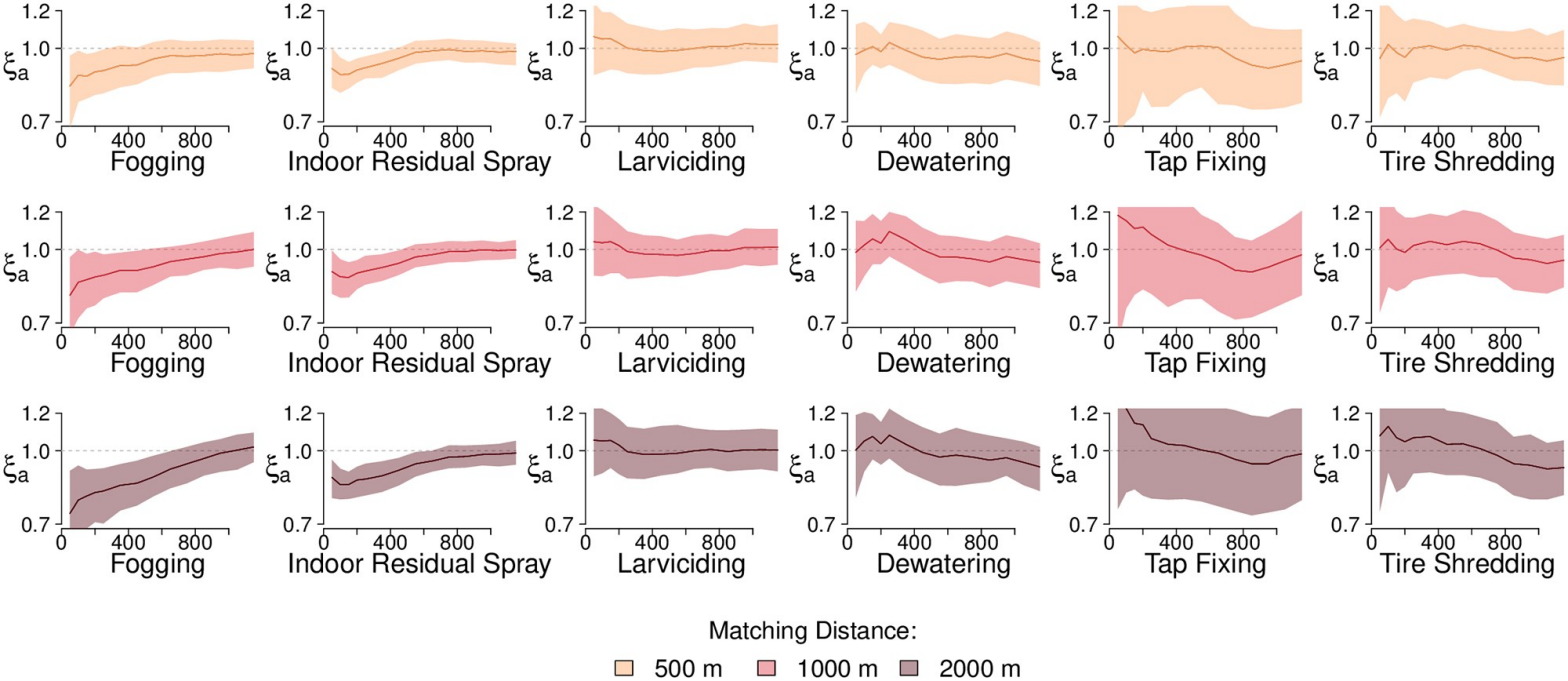
Variation in the effect of containment activity versus the distance (in meters) from index cases. Variation in the effect of containment activity, *ξ*_*a*_(*d*_1_, *d*_2_), versus the distance (in meters) from index cases using data from Rawalpindi and Lahore across various values of matching distance *m*. Values of *ξ*_*a*_ are calculated using containment and non-containment cases which appear in an *m* = 1000 m radius of each other. The spatial window of the analysis (*d*_2_ minus *d*_1_) is maintained at 500 m when *d*_2_ is greater than 500 m, and observations are made by sliding the window at intervals of 100 m. For *d*_2_ less than 500 m, *d*_1_ is equal to zero and observations are made by increasing *d*_2_ at intervals of 100 m. Spatial dependence estimates are plotted at midpoint of the spatial window. Values below 1 show a lower probability of new cases appearing around a case in proximity of a containment activity, compared to a non-containment case. The time window *t*_2_ − *t*_1_ is set to 30 days. 95% CI from bootstrapping 100 replications are shown as shaded areas around estimates.

For each containment case we identified a matching non-containment case with a similar epidemiological context (in spatio-temporal proximity) but did not have a containment activity performed in its immediate spatio-temporal vicinity. We then calculated the reduction in probability of generation of new cases, *ξ*_*a*_(*d*_1_, *d*_2_): the ratio of the spatial dependence of containment to that of matching non-containment cases (Methods; Fig 3). Values of *ξ* below 1 indicated a reduced probability of observing new cases around cases which received a containment activity compared to matching non-containment cases. Sensitivity analysis for varying spatial and temporal windows showed that results are not sensitive to the spatial and temporal window used to match non-containment cases (see Fig B-D in S1 Appendix for sensitivity analyses results).

We observed a 0.9 times reduced probability (10% reduction) of a case occurring anywhere from distance *d*_1_ = 0 m to *d*_2_ = 100 m, and in the next 30 days of cases which had IRS performed in the immediate vicinity (95% CI: 0.81–0.99). For fogging, this value was 0.80 (95% CI: 0.66-0.96). By distance range of *d*_1_ = 500 m to *d*_2_ = 1000 m for IRS and range *d*_1_ = 800 m to *d*_2_ = 1300 m for fogging, there was no difference (*ξ*_*a*_ = 0.99) in probability of new cases around the containment and non-containment cases (Fig 3; see Table C in S1 Appendix for *ξ*_*a*_ values at different distances for each containment activity). In contrast to fogging and IRS, resulting plots for other activities indicate structural uncertainty. This is due to the low number of cases in proximity of these activities, and thus our results indicate no conclusive relationship between deployment of activities other than fogging and IRS and reduction in probability of new cases. Results are summarized in Table D in S1 Appendix.

### Containment activities in relation to *R*_0_

The model for each city was fit with and without containment activities data to identify the improvement in fit of the reported case data by addition of containment activities in the model, providing a more realistic model of dengue transmission in our scenario.

The model trained on case data by spatial unit in Rawalpindi, without containment activities (only environmental parameters and population density), provided a good fit with reported cases from the spatial units (adjusted *R*^2^ 0.78; Akaike information criterion (AIC) 83018.46), and addition of containment activities to the model improved the fit (adjusted *R*^2^ 0.81; AIC 79638.58). Similarly, for Lahore, the model incorporating containment activities improved the fit (adjusted *R*^2^ from 0.73 to 0.76; AIC from 114049.20 to 109136.30). During the peak dengue activity season, between August and November, we observed a reproductive number of 2.92 (at a mean temperature of 28.6 Celsius and rainfall for 4 days during a 2 week period) in Rawalpindi, and 2.23 (at a mean temperature of 30.0 Celsius and rainfall for 3 days during a 2 week period) in Lahore. Across the spatial units in Rawalpindi we observed a median mixing parameter, *α*_*i*_, of 0.78 (min: 0.64, max: 0.88, *n* = 14), while in Lahore the median mixing parameter was 0.83 (min: 0.77, max: 0.90, *n* = 10) (Methods). Values of mixing parameter closer to 1 represent more homogeneous mixing of infected and susceptible individuals. Incorporating this parameter in the model allows for accounting of variation in population mixing across each spatial unit, allowing for more realistic modelling of dengue transmission. We also found increases in average temperature and rainfall days, during a time step (2 weeks), to be related to increases in *R*_0_, consistent with previous work [34]. Increases in population density were also related to increases in *R*_0_, also as expected [47].

**Fig 4:**
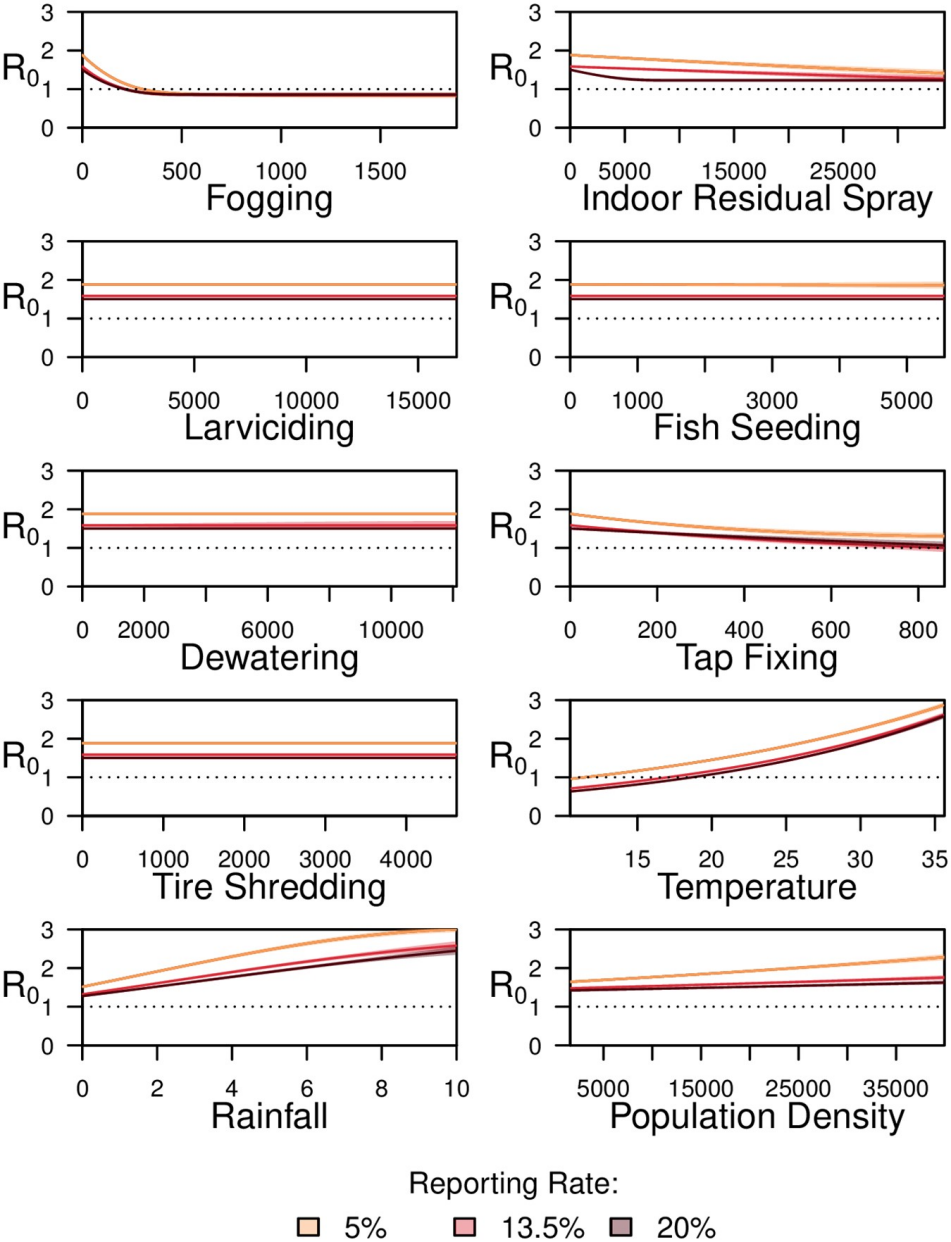
Variation in reproductive number (*R*_0_) of dengue, with variation in parameters, in Lahore. Variation in reproductive number (*R*_0_) of dengue, with variation in parameters, in Lahore during 2012–2017 for different incidence reporting rate values. For containment activities, x-axis represents the total number of containment activities performed, in a spatial unit, in a lagged time step and any residue from previous weeks. For temperature, x-axis represents the average temperature in Celsius in a lagged time step. For rainfall, x-axis represents the number of rainfall days in a lagged time step. For population density, x-axis represents individuals per 1 sq.km.

**Fig 5:**
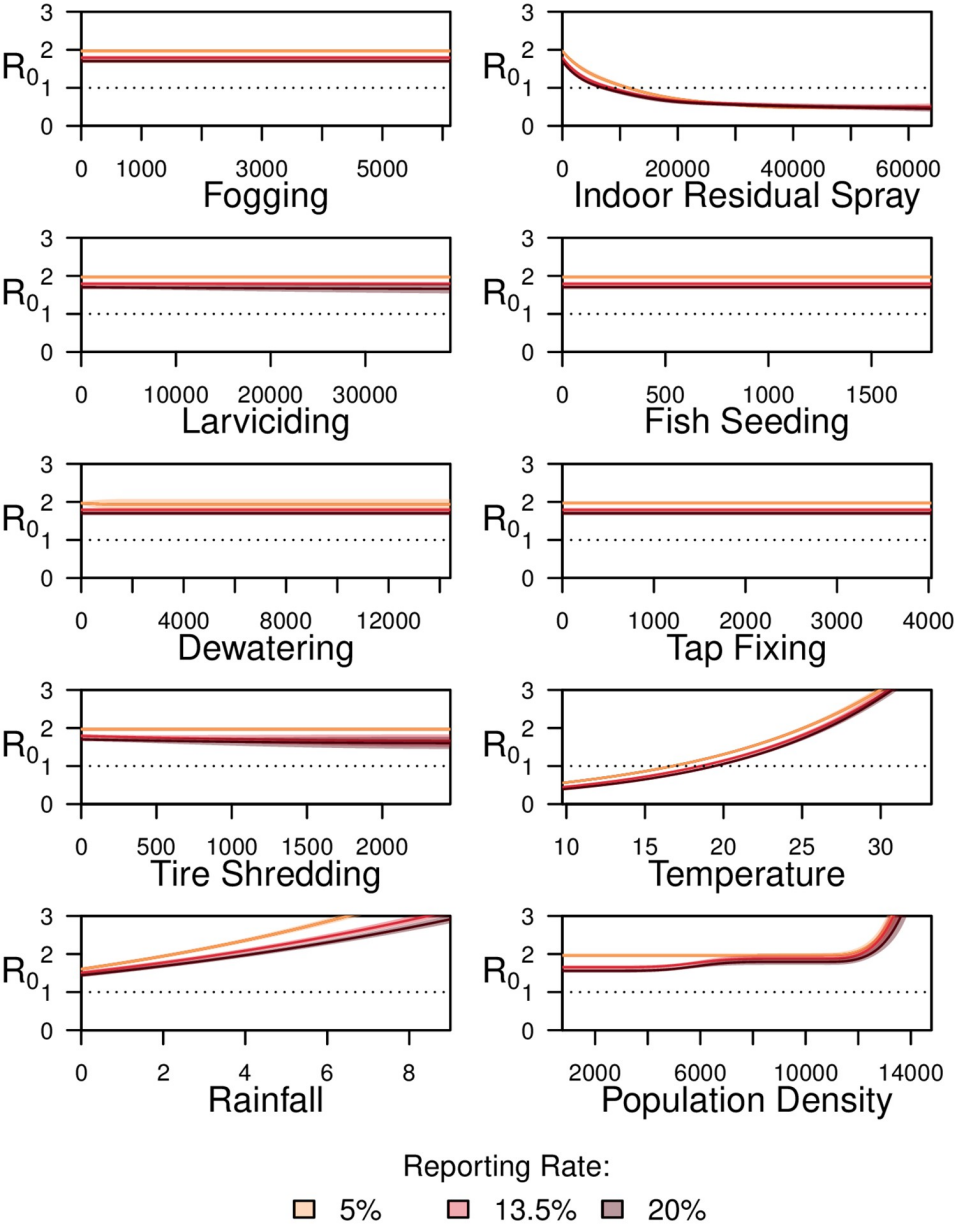
Variation in reproductive number (*R*_0_) of dengue, with variation in parameters, in Rawalpindi. Variation in reproductive number (*R*_0_) of dengue, with variation in parameters, in Rawalpindi during 2014–2017 across various incidence reporting rate values. For containment activities, x-axis represents the total number of containment activities performed, in a spatial unit, in a lagged time step and any residue from previous weeks. For temperature, x-axis represents the average temperature in Celsius in a lagged time step. For rainfall, x-axis represents the number of rainfall days in a lagged time step. For population density, x-axis represents individuals per 1 sq.km.

Findings illustrate varied relationships between the amount of containment activities applied and the *R*_0_ (Figs 4 and 5). Of the adulticides, we found an increase in IRS to be related to a decrease in *R*_0_ of dengue in both Lahore and Rawalpindi. As modeled, in Rawalpindi, with an application of 8354 (95% CI: 7453–9394) IRS activity instances, the *R*_0_ reduces below 1. In Lahore the maximum number of IRS activity instances deployed during any single time step were 34270, at which *R*_0_ decreases to 1.27 (95% CI: 1.19–1.36) according to modelling results. All results described here are under mean temperature, rainfall and population density, while controlling for the effect of other containment activities (Methods). In contrast, increases in fogging was were related to decrease in *R*_0_ only in Lahore; with the deployment of 222 (95% CI: 199–252) instances of containment the *R*_0_ reduces below 1. Upon closer inspection, we found that, over the time period of the study, the number of IRS and fogging containment activity instances were highly correlated in Rawalpindi (Pearson’s correlation of 0.74), as opposed to in Lahore, (correlation of 0.06). Thus, due to the multicollinearity of IRS and fogging activities, in the model for Rawalpindi no conclusive relationship between fogging and *R*_0_ of dengue in Rawalpindi should be drawn.

Among source reduction activities, tap fixing was related to a decrease in *R*_0_ in Lahore only, and the maximum number of activity instances deployed during a single time step were 860, at which the *R*_0_ reduces to 1.0 (95% CI: 0.9–1.11) per the model. For the remaining activities targeted at source reduction and at the larval stage of mosquitoes, we could not draw conclusions regarding significance of their relationship with *R*_0_. These relationships, between containment activities and *R*_0_, are not sensitive to the reporting rate. Results are summarized in Table E in S1 Appendix.

## Discussion

Containment activities play an integral role in reducing the burden of dengue [8]. Though numerous previous studies have assessed the relationship between containment activities and epidemiological indicators, there is lack of evidence on the localized relationship between containment activities and dengue incidence in real-world settings [16, 17]. Leveraging millions of data points on seven different vector containment activities each linked to precise geo-coordinates, from a containment activities monitoring system, deployed in Pakistan, we develop two frameworks and use them to analyze the effect of containment activities on dengue incidence, in space and time. The analyses are designed in light of the non-random and highly localized nature of containment activity deployment, which is often the case in acute situations such as outbreaks. Thus besides the specific findings regarding containment activity efficacy, this study also provides important lessons regarding modeling limitations of real-world (observational) data that can inform future intervention or containment activity deployment.

First, we examined the relationship between deployed instances of each containment activity type and the spatial dependence of geo-located dengue cases in their proximity, in the cities of Rawalpindi and Lahore. This method allowed us to control for exogeneous heterogeneities that could create spatial or temporal clustering (e.g., biases in where the activities were deployed are minimized as non-containment cases which are compared, are chosen in the immediate space and time vicinity of cases which had containment activities deployed next to them). The result is measurement of the maximum reduction in dengue transmission in the vicinity of a particular type of activity, as well as the maximum distance at which this reduction in dengue transmission is evident. Notably, the method and results provides novel empirical results and insights into the comparative relationship of fogging and indoor residual spray with real case data. Specifically, these results are the first to show how the reduction in generation of new cases changes as a function of distance from where fogging and IRS were applied. The methods also provide a way to quantify the maximum distance up to which the reduction is present, which can help officials optimize the deployment of these containment activities. Similarly, results from the time series modeling approach are based on empirical field data (opposed to using simulated data or proxies) and consider multiple containment activities while directly accounting for external factors such as environmental parameters and population density. The analysis also uses a precise and standardized measure of efficacy (*R*_0_), in contrast to using proxy measures for dengue transmission [15].

Spatial dependence of dengue cases reported here is consistent with that reported in previous work using dengue case data from Bangkok (dependence at 200 m was 1.82 (95% CI: 1.45–2.16), comparable to 1.83 and 1.45 observed in Rawalpindi and Lahore) [29]. Lower spatial dependence in the city of Lahore, as compared to Rawalpindi, is consistent with the fact that the city faced a major dengue outbreak in 2011, which would have increased immunity in the population. In contrast, the city of Rawalpindi faced a less severe outbreak in 2011, hence immunity in the population there would be lower [29].

From time series modeling, we observed an average *R*_0_ value of 2.92 in Rawalpindi and 2.23 in Lahore, during peak dengue activity season in Pakistan (August–November). These values are in line with those reported in previous work in Pakistan, which shows that the average *R*_0_ values during these months, across all cities of Punjab (of which both Rawalpindi and Lahore are situated in), vary roughly between 1 and 3 [24]. The *R*_0_ values of dengue reported here are also in line with *R*_0_ values reported for dengue in other countries including an analysis of nine outbreaks in Brazil, between 1996–2003, five of which reported *R*_0_ values equal to or less than 3 [48]. We also observed a median mixing parameter of 0.78 in spatial units in Rawalpindi and 0.83 in spatial units Lahore. Previous dengue research in Pakistan has reported a mixing parameter of 0.74 for the city of Lahore, overall [24]. This value is lower than the median value observed in our study, given that our work computes this parameter at a sub-city spatial resolution as opposed to city-level spatial resolution which would be assessing mixing on average. In addition, mixing parameters are very specific to the population of a location, and we are not able to make a direct comparison given that we did not find any previous study which models the transmission of dengue at a sub-city level in Lahore or Rawalpindi.

Results from the spatial signature analysis show that IRS and fogging spray, in the vicinity of a dengue case, result in lower probability of new cases by 10% (95% CI: 1-19%) and 20% (95% CI: 4-34%) respectively. Additionally, IRS and fogging are shown to be associated with a reduced probability of new cases in their vicinity (*ξ*_*a*_(*d*_1_, *d*_2_) below 1) up to a distance of 750 m (±250 m; *d*_1_ = 500 m, *d*_2_ = 1000 m) and 1050 m (±250 m; *d*_1_ = 800 m, *d*_2_ = 1300 m) respectively. Similar trends are observed based on the results of time series modelling of containment activities; increases in IRS and fogging are related to decreases in the reproductive number of dengue in Lahore, though results from Rawalpindi only show a significant reduction in *R*_0_ for IRS from those activities aimed at the adult phase of the mosquito lifecycle.

We could not draw conclusions about activities aimed at the larval stage of the mosquito lifecycle in this study. However, it should be noted that lack of evidence of a significant relationship between activities and *R*_0_, or spatial dependency of cases, does not conclude that no relationship exists. Previous studies, summarized in a recent systematic review, found Temephos (a chemical used in larviciding) to be only effective in reducing entomological indicators [16]. However, this review did not generate any evidence of its association with reduction in disease transmission. Moreover, this previous work had been performed under laboratory conditions, and while containment activities can be effective or ineffective under laboratory conditions, the effect may not translate exactly in the field in reducing dengue transmission. In our case, there may not have been enough cases with solely larviciding in their vicinity, at the relevant times, or enough larviciding happening near cases where it did occur, in order to show efficacy. Both our findings, and gaps in previous work, signify the important utility of studies such as this which examine the result of containment activities using empirical disease incidence data and containment activity deployment. Based on our lack of significant results for larviciding, we can conclude that there would be a need to deploy more containment activities in the field, in order to assess efficacy. This is also the case for fish seeding. There is conflicting evidence regarding the effectiveness of fish seeding in the literature [49, 50]. As well, based on deployment of this activity in our dataset, the time series method did not find evidence of a significant relationship between increase in fish seeding activity and reduction in *R*_0_ in either city. In addition, no conclusion about fish seeding could be drawn from the spatial dependence method based on the number of activities performed in our dataset. Therefore, more cases which were adjacent to only fish seeding activities, or more fish seeding per adjacent case would be required in the real-world setting to robustly assess this containment activity.

Among source reduction containment activities, we found an increase in tap fixing in Lahore to be associated with a statistically significant decrease in the reproductive number of dengue in Lahore. This result may be attributed to leaking taps being a constant source of fresh water, which provide a breeding ground for Aedes aegypti mosquitoes [51]. A small number of previous studies have discussed water supply issues. While these generally were positive on the importance of correcting such challenges, they did not directly assess the effect of addressing water supply issues [52], or only looked at the aggregate effect of such an activity along with other activities [53]. The time series or spatial dependence approaches did not provide evidence of a significant relationship between other source reduction activities and dengue incidence. Again, either inefficacy of the activities, sparsity of cases which had source reduction activities performed in their vicinity, or the amount of source reduction per case, could be the reason(s) results from the spatial dependence analysis were inconclusive. Given difficulty in drawing conclusions for some activities, further in this section we provide recommendations to ensure future, pragmatic deployments can enable more systematic analyses.

As is the case and is a common challenge in SIR modeling research, spatial granularity of the analysis is important to ensure that assumptions regarding transmission, and in this case, efficacy of interventions, are modeled appropriately. Here, containment activities considered should be physically relevant to the cases in the same spatial unit. This would mean that they are uniformly distributed with respect to cases (to ensure similar epidemiological context), and given the immediate spatial impact of the containment activities considered, these should be as close as possible to the cases as well. If these assumptions are violated, then resulting findings from the model may not be completely accurate [18, 54]. In this study, we minimize heterogeneity and increase relevance between cases and containment activities by studying the relationships at a relatively small, sub-city spatial units, as opposed to at a city level as done in previous work [24, 36]. Additional steps, such as including higher resolution environmental data and landscape features, and socio-economic factors in the modelling approach, where available, can be taken, to further reduce potential biases. There are additional factors, such as movement of humans within a city, which can potentially introduce biases in results. Given the lack of reliable mobility data, we did not account for movement of individuals in the study. Although other recent SIR modeling analyses have not incorporated mobility [24, 55], if such data were available at the sub-city level in Punjab it could potentially be useful. Although, a major difference is that our study would require sub-city movement data, the relevance of which would have to be examined in disease transmission, as existing studies incorporating mobility have done so at a coarse spatial resolution (e.g. between districts or across an entire nation) [56, 57].

Given that the containment activities are often not systematically deployed, but instead must be deployed pragmatically by public health agencies during an outbreak, the data is thus observational in nature and there could be a possibility of external biases. In our study we mitigate such biases by using a spatial dependence metric which accounts for underlying spatial and temporal clustering of cases and by modeling the containment activities and cases at a sub-city level. Further, while we consistently observe a short-term positive impact of IRS on dengue incidence, we cannot rule out these containment activities simply delay infection to future time points [58]. Given the data at hand, we were unable to assess the longer-term impact of the containment activities. Due to close proximity of cases within the city and the necessity to deploy containment activities during an outbreak, we could not isolate a sufficient number of cases which only had a single containment activity in their vicinity before symptoms were reported for that case, and no containment activity afterwards. Moreover, it should also be noted that results from this study are only relevant to the spatial dependence of cases or relationship between containment activity deployment and *R*_0_ after dengue cases have started to appear. Results from the study do not explain the preventative relationship of a containment activity, i.e. when no cases are being reported in an area. Specific to the spatial signature analysis there are additional requirements, which include containment activities being in very close proximity of a case and presence of only a single containment activity in the proximity of a case.

Quantifying the impact of interventions requires detailed data on when and where interventions are deployed. We use this opportunity to make recommendations for future deployments of containment activities and data collection requirements that could enable more systematic analyses, while still allowing for pragmatic deployment based on the priorities of a government or public health department. First, even in an outbreak situation, containment activities should be deployed in a systematic manner throughout a community and throughout the dengue season, by ensuring that the amount of containment per case is consistent across space and time. This would require assessing the distribution of containment and cases at multiple time points during an outbreak or season, but would enable a more even assessment across all cases. Second, alongside information on the location, timing and type of interventions, public health authorities should record whether activities were deployed proactively (in anticipation of cases occurring), as opposed to reactively (in response to cases occurring) to better understand temporal ordering of cases and activities and enable clearer study of prevention versus containment effects. Third, also towards being able to study the preventative effect of containment activities (which we were not able to do here, given the data), containment activities could be performed even in areas which have no cases, to study if the application of containment activities relates to a delay in the appearance of new cases. This would allow systematic comparison between appearance of first cases in areas which received containment, in comparison to similar areas which did not. Fourth, it would be helpful to record places where individuals have spent significant time in the two weeks prior to developing dengue symptoms, especially outside the home. This would help identify locations where potential for infection is high but would not be captured by analyses based on household locations only. While following all of the mentioned recommendations might not be practical, especially in a real-world setting, based on modeling experience here such strategies can help perform more robust comparisons of the effectiveness of containment activities.

More broadly, these results based on real-world observational data—and the models and methods used to derive them—are relevant to a growing number of global health concerns related to the *Aedes aegypti* mosquito, including the Zika virus and chikungunya, which are also known to particularly impact urban areas. The methods presented in the work lay important groundwork for future prospective and retrospective studies aimed at using systematic data collection methods to obtain robust measures of the efficacy of containment activities from empirical data.

## Supporting information

S1 Appendix Extended methods, results, sensitivity analyses and discussion. (PDF)
