# Supplementary material for: Quantifying the localized relationship between vector containment activities and dengue incidence in a real-world setting: A spatial and time series modelling analysis based on geo-located data from Pakistan
Source: PLoS Negl Trop Dis. 2020 May 11;14(5):e0008273. doi: 10.1371/journal.pntd.0008273 (PMC7241855; doi:10.1371/journal.pntd.0008273)
Supplement: S1 Appendix — (PDF) [file pntd.0008273.s001.pdf]

Quantifying the localized relationship between vector containment activities and dengue incidence in a real-world setting: a spatial and time series modelling analysis based on geo-located data from Pakistan

Nabeel Abdur Rehman, Henrik Salje, Moritz U G Kraemer, Lakshminarayanan Subramanian, Umar Saif, Rumi Chunara

Appendix

1. Background of Study Setting and Disease Reporting

The province of Punjab, in the north of Pakistan, provides a unique opportunity to study the spread of dengue and understand the impact of containment efforts in a real world setting. In Pakistan, the transmission of dengue viruses was largely confined to the southern city of Karachi until 2011 when a large dengue epidemic with over 20,000 cases occurred in the northeastern city of Lahore, causing significant morbidity and mortality and established this region of the country as the focus of seasonal dengue epidemics [1, 2]. Since 2012 Lahore has subsequently seen two major outbreaks, in 2013 and 2016. Similar to Lahore, the city of Rawalpindi has seen an increased dengue activity in the region since 2011 with subsequent dengue outbreaks since end of 2013. The magnitude of outbreaks in Rawalpindi has been higher as compared to Lahore.

Lahore is the provincial capital and the most populous city of Punjab, while Rawalpindi is the third most populous city of the province. Reporting has been upheld and promoted in both cities per numerous factors described below, which allow us to assume that reporting is consistent across space and time. First, it should be noted that as the city of Lahore houses over 100 hospitals while the city of Rawalpindi houses over 50 hospitals. Hence issues related to patient travel are negligible. All public hospitals report dengue patients' details, including date of admit, onset date of fever, and home address to a central server for easy data collection. While there are government personnel based at every public hospital to report the data, the private hospital are asked to report the same data but do so minimally (see Reporting Rate section in this SI). The data from both private and public hospitals is entered into a central server by hospitals on a daily basis. Given that health care in public hospitals is free, the cost of treatment is not a limitation for patients either. Fixed guideline criteria are followed by all hospitals which supports consistent diagnoses across all hospitals. The criteria are described subsequently in this appendix.

2. Smartphone Application

The Punjab Information Technology Board (PITB) has a recent history of innovation in disease monitoring systems; for example a working health hotline with a widely spread toll free number allows people to get advice about disease without leaving their homes [3]. Traditionally, containment activities performed in Pakistan were monitored by local public health departments using paper-based records generated by health workers. This protocol had several drawbacks. First, there was no physical evidence that the containment activity had indeed been performed by the worker; instances occurred wherein workers provided falsified reports of activities that were never carried out. Second, paper forms were often lost or arrived at the centralized compilation unit with inconsistencies and delays.

In January 2012, after the intense epidemic in 2011, under the supervision of the PITB, smartphones were distributed amongst health workers, in the province (predominantly in Lahore and Rawalpindi), to replace the paper-based recording system described. The phones had a pre-installed application (the Punjab Anti Dengue Activity Tracking System) for tagging and reporting containment activities. The application was designed in the native Urdu language, with an intuitive interface that catered to the semi-literacy of the workers (Fig. A). Seminars were carried out by the Punjab Information Technology Board to both familiarize workers with the use of the application and to provide specific guidelines to carry out each containment activity in an effort to ensure consistency. Once implemented, workers were required to use the application to document containment activities by taking pictures of the location both before and after performing the activity, and they were required to fill in a short form describing details of the activity. Pictures of before and after performing containment activity allowed the government to ensure that the desired containment activity had been performed. Global positioning system (GPS) coordinates of the location, time stamp, details and pictures of the performed activity were then automatically submitted to a centralized server. Health workers were given government authorized Subscriber Identity Module (SIM) cards, for free use of telecommunication service when performing containment activities.

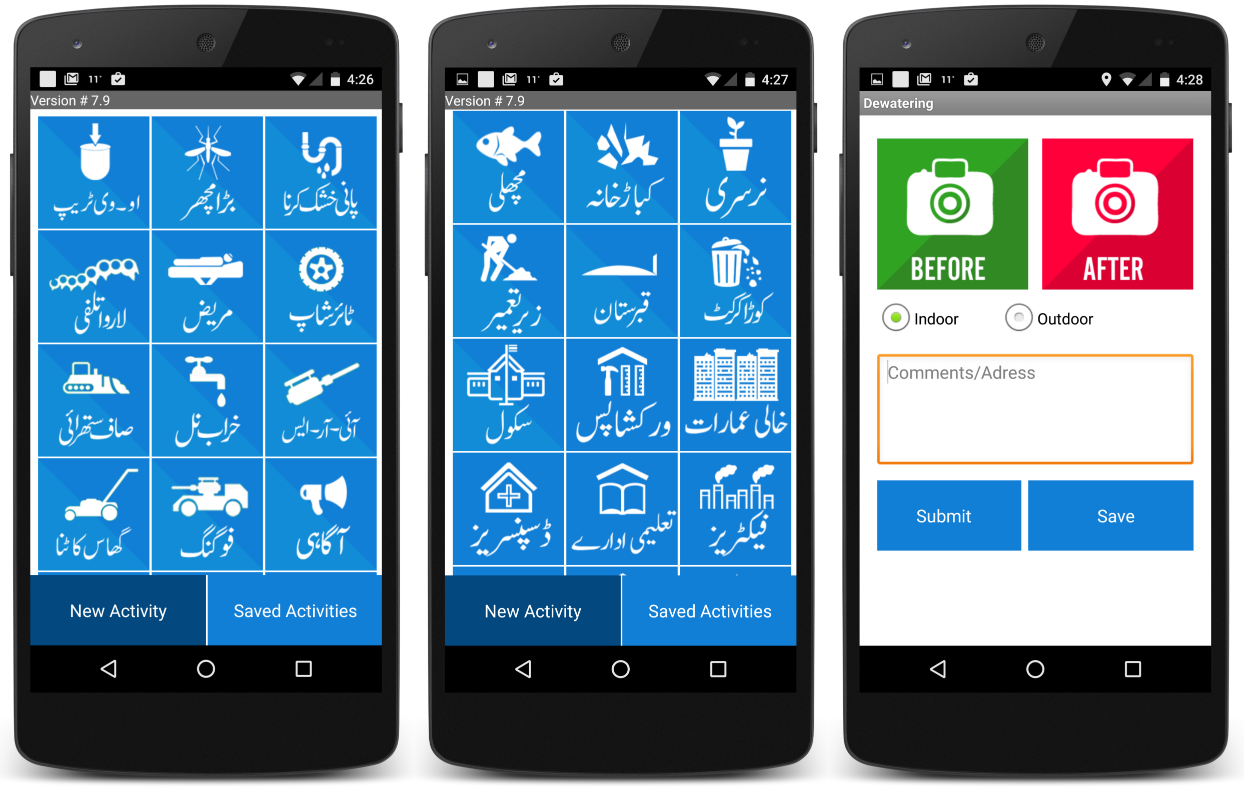

Figure A: Screenshots of the dengue containment activity tagging application.

Table A: Total number of containment activities captured by the system (2012–2017), captured in Lahore (2012–2017) and in Rawalpindi (2014–2017), by activity type.

Activity	All Locations (2012–2017)	Lahore (2012–2017)	Rawalpindi (2014–2017)
Dewatering	2275903	637950	261413
Fish Seeding	92996	30154	4190
Fogging	188316	33003	109137
IRS	1314043	562798	456298
Larviciding	1745163	552871	648992
Tap Fixing	359961	41867	53662
Tire Shredding	1305550	507575	77249

3. Containment Activities

Dengue containment activities are broadly grouped in 3 categories [4]:

- Source reduction (location and destruction of mosquitoes' breeding sites)
- Use of larvicides to kill mosquitos at the larvae stage
- Use of ultralow-volume aerosolized adulticides to kill adult mosquitos

The Punjab Anti Dengue Activity Tracking System app provides an interface to tag activities related to all of the above three strategies. Source reduction activities and activities targeted at larval stage are carried out throughout the year, focusing on reducing the breeding and development sites, although their frequency is increased during peak dengue activity season. Daily surveys of locations, randomly assigned to them, are performed by health workers and source reduction or activities targeted at larval stage are performed if a potential dengue vector breeding or development site is discovered. In contrast, adulticide activities are largely performed during the high dengue activity season, and are specifically targeted in areas where patients have already started to appear. This is due to the fact that these activities involve chemical control, are costly, and can lead to resistance of chemicals in the dengue vector [5]. The ad hoc nature of deployment of these containment activities require systematic analyses of the data. We describe below the details of the containment activities, included in our study, categorized by the stage in the life cycle of mosquitoes they are targeted. While additional containment activities were performed in both cities, due to lack of complete and continuous reporting of such activities, we included the following in this study.

3.1. Source Reduction Activities

3.1.1. Dewatering

Dewatering is the removal of stagnant water, which is either already a breeding ground or has a potential to turn into a breeding ground soon. The water is generally polluted and often accumulates after rainfall or overflow of sewage pipes. The activity is carried out by the water and sanitation agency (WASA) in Pakistan.

3.1.2. Tap/Water Leakage Fixing

Water leakage from public taps and fountains often accumulates in a small pond that can act as a breeding ground of *Aedes aegypti* mosquito. This tag is used to show if a worker fixed a leak.

3.1.3. Tire Shredding

Old tires are convenient breeding grounds for *Aedes aegypti* mosquitoes as they go unnoticed and water can stay inside old tires for a long time. During routine surveys, tires in automobile repair shops with accumulated water are drained.

3.2. Larvae Control Activities

3.2.1. Applying Larvicide

During routine surveys, containers of *Aedes aegypti* larvae are drained and treated with Temephos 50 EC @ 16ml/10 liter [6]. Entomologists often accompany field workers during these surveys to identify *Aedes aegypti* larvae.

3.2.2. Fish Seed

Large ponds which are difficult to drain, or are breeding sites for fish are treated by introducing Tilapia fish. The fish are known to eat larvae of mosquitoes. The activity is carried out by the fisheries department in Pakistan.

3.3. Adulticide Activities

3.3.1. Fogging

Fogging is the mass anti-dengue spray that is carried out in open places in order to kill adult mosquitoes. A single fogging spray activity represents spraying a neighborhood of 50 houses. Deltamethrin 1.5 EC @ 33ml/5 liter of diesel is used for fogging spray [7] .

3.3.2. Indoor Residual Spray

The indoor residual spray is deployed indoors and sticks to the walls to kill the *Aedes aegypti* mosquitoes. A single IRS activity represents spraying of a total of 50 houses. Deltamethrin 5 WP @ 125gm/10 liter of water is used for IRS spray [7].

4. Patient Portal and Criteria for Patients

Data entry personnel are assigned to each public government hospital, who are responsible for reporting the details of dengue patients into the centralized portal managed by the Punjab Information Technology Board. Dengue cases reported by the hospitals are categorized into three types: suspected, probable and confirmed. The criteria used by hospitals to determine a suspected dengue cases is: fever for 2 – 10 days with two or more of the following symptoms: retro orbital pain, myalgia, arthralgia/severe backache/bone pain, rash, bleeding manifestations (epistaxis, hematemesis, bloody stools, menorrhagia, hemoptysis), abdominal pain, decreased urinary output despite adequate fluid intake and irritability in infants. The suspected dengue cases are further tested and are categorized as probable if lab results show both of the following:

- Thrombocytopenia = 100,000/mm^3
- Leukopenia = 4,000/mm^3

The probable dengue cases are further tested using viral test for confirmation of dengue infection. The criteria used by hospitals used to determine a confirmed dengue viral infection assessed via at least one of the following:

- Detection of viral antigen (NS-1 antigen in blood)
- Detection of virus by PCR
- Detection of IgM
- Demonstration of = 4 folds rise in IgG antibody titre in paired acute and convalescent serum

Laboratory tests for IgM, IgG and NS-1 were carried out by ELISA using a kit which covers all four strains of dengue virus (DEN 1 - 4). For our study, we use only confirmed dengue cases data as it includes the actual number of dengue infections.

Starting in 2014, to acquire geo-location information for the cases, each confirmed dengue patient entered in the portal was assigned a unique identification number. The details of the confirmed patients were made available in the smartphone application. The containment workers were then designated to geo-tag the home locations of the confirmed patients. Hence, in our dataset, all confirmed cases in Rawalpindi and Lahore had a point coordinate associated with them (data starting from 2014), and data prior to 2014 from Lahore, while not having a precise geo-location, had a linked spatial unit.

While each patient's home address was precisely tagged with geo-coordinates using smartphones during data collection; for the spatial analysis in this study, for both containment and incidence data, we accessed transformed geo-location coordinates to avoid privacy concerns (spatial analysis). The original geo-located latitude and longitude coordinates were transformed to the UTM (Universal Transverse Mercator) coordinate system and had a random offset added. This ensured that the relative distance between individual data points was unchanged, while also obscuring the original coordinates. For the time-series analysis we accessed containment and case data aggregate to the spatial unit level (described in "Time-series modelling" section).

For both the time-series and spatial dependence analysis, for consistency, the date of fever-onset of a case is used instead of the reporting or the confirmation date of the case. This was done due to the fact that confirmation date of dengue case can vary from weeks to a month from actual infection date, and depends on when a patient arrives at the hospital (reporting date) and how long it takes for the laboratories to run the tests.

5. Spatial Signature - Extended Results

5.1. Sensitivity to Distance and Time Windows Used

To identify if the inferred impact of containment activities varies based on the selection criteria of matching containment cases with non-containment cases, we calculate ξ_a for different matching distances m (500, 1000 and 2000 meters). Fig. 3 (main text) shows the effect of containment activities for different values of m. As evident from the results, in general, both fogging and IRS decrease the likelihood of new cases appearing around cases which were in proximity of the activity. We find that as the value of m increases, the probability of new cases around those which were in proximity of IRS or fogging as compared to around non-containment cases decreases even further. Moreover, this decrease in the probability of new cases is larger for fogging as compared to IRS. Additionally, we also vary the time window $t_2 - t_1$ of our study to identify if the results are sensitive to the time window. The relative probability of new cases around those in proximity of fogging or IRS in this case does not change with the time window used (Fig. C).

Table B: Total number of cases, per containment activity used in our analysis, and the number of cases which had a matching non-containment case (m=1000m), for Rawalpindi and Lahore

	Rawalpindi		Lahore	
Case Type	Total cases	Cases with a matching non-containment case	Total cases	Cases with a matching non-containment case
Fogging	155	152	7	7
IRS	512	508	19	17
Larviciding	192	182	83	73
Tap Fixing	23	23	2	2
Tire Shredding	50	49	33	25
Dewatering	102	101	7	5
Non-containment	6021	–	1157	–

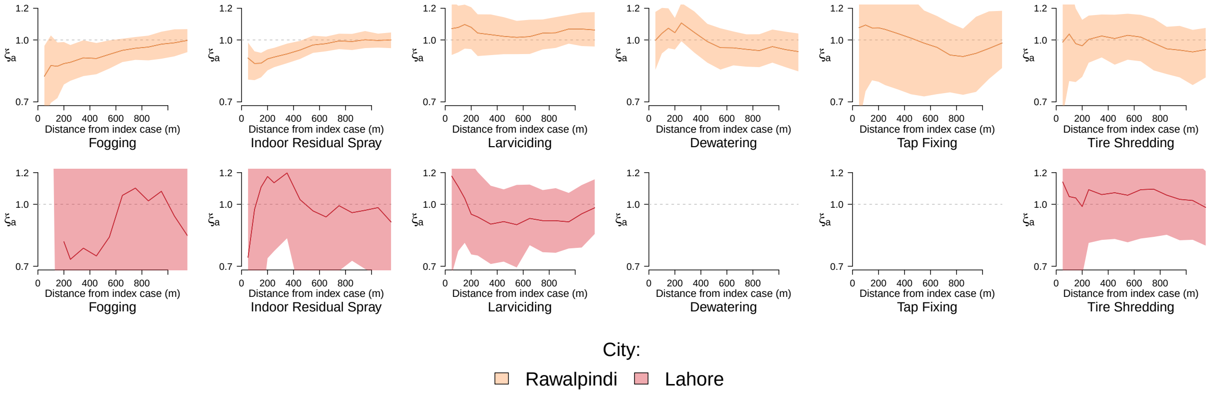

Figure B: Variation in the effect of containment activity, ξ_a, versus the distance (in meters) from index cases in Rawalpindi and Lahore separately. Values of ξ_a are calculated using containment and non-containment cases which appear in an m=1000 m radius of each other. The spatial window of the analysis (d_2 minus d_1) is maintained at 500 m when d_2 is greater than 500 m, and observations are made by sliding the window at intervals of 100 m. For d_2 less than 500 m, d_1 is equal to zero and observations are made by increasing d_2 at intervals of 100 m. Spatial dependence estimates are plotted at midpoint of the spatial window. Values below 1 show a lower probability of new cases appearing around a case in proximity of a containment activity, compared to a non-containment case. The time window $t_2 - t_1$ is set to 30 days. 95% CI are shown as shaded areas around estimates. For Lahore, several plots indicate structural uncertainty and very large confidence intervals, though mean values are visible for the entire range of activities considered.

Table C: Values of ξ_a for each containment activity, with varying distance from index cases for m= 1000 meters.

Distance (meters) $(d_1+d_2)/2$	d_1–d_2 Range (meters)	Fogging	Indoor Residual Spray	Larviciding	Dewatering	Tap Fixing	Tire Shredding
50	0–100	0·8 (0·66,0·96)	0·9 (0·81,0·99)	1·04 (0·88,1·24)	0·99 (0·82,1·15)	1·18 (0·63,1·84)	1·01 (0·74,1·38)
100	0–200	0·85 (0·72,1)	0·88 (0·79,0·95)	1·03 (0·88,1·21)	1·02 (0·88,1·15)	1·15 (0·75,1·67)	1·05 (0·83,1·3)
150	0–300	0·86 (0·75,0·98)	0·87 (0·79,0·94)	1·04 (0·89,1·17)	1·05 (0·93,1·19)	1·11 (0·8,1·47)	1 (0·82,1·2)
200	0–400	0·88 (0·76,0·99)	0·89 (0·83,0·95)	1·02 (0·89,1·13)	1·03 (0·93,1·15)	1·11 (0·82,1·46)	0·99 (0·83,1·17)
250	0–500	0·88 (0·79,0·98)	0·9 (0·84,0·97)	0·99 (0·87,1·09)	1·09 (0·97,1·2)	1·08 (0·81,1·43)	1·02 (0·86,1·17)
350	100–600	0·9 (0·81,0·98)	0·92 (0·86,0·98)	0·98 (0·87,1·07)	1·05 (0·93,1·17)	1·02 (0·76,1·34)	1·04 (0·88,1·19)
450	200–700	0·9 (0·81,0·99)	0·94 (0·88,1)	0·98 (0·88,1·07)	1 (0·88,1·11)	0·99 (0·78,1·28)	1·02 (0·89,1·18)
550	300–800	0·92 (0·84,1·01)	0·96 (0·92,1·03)	0·97 (0·88,1·05)	0·97 (0·85,1·07)	0·97 (0·79,1·18)	1·04 (0·89,1·21)
650	400–900	0·94 (0·88,1·01)	0·98 (0·93,1·03)	0·98 (0·89,1·07)	0·96 (0·86,1·07)	0·94 (0·74,1·14)	1·03 (0·88,1·19)
750	500–1000	0·95 (0·88,1·02)	0·99 (0·94,1·04)	0·99 (0·9,1·08)	0·96 (0·86,1·05)	0·9 (0·71,1·06)	1 (0·87,1·15)
850	600–1100	0·97 (0·91,1·04)	0·99 (0·94,1·04)	0·99 (0·91,1·07)	0·94 (0·83,1·04)	0·9 (0·72,1·04)	0·96 (0·82,1·09)
950	700–1200	0·98 (0·92,1·06)	1 (0·95,1·04)	1·01 (0·93,1·11)	0·97 (0·86,1·07)	0·92 (0·74,1·09)	0·95 (0·81,1·07)
1050	800–1300	0·99 (0·91,1·07)	1 (0·95,1·04)	1·01 (0·92,1·1)	0·95 (0·84,1·05)	0·95 (0·77,1·14)	0·93 (0·81,1·05)
1150	900–1400	1 (0·92,1·09)	1 (0·96,1·05)	1·01 (0·93,1·11)	0·94 (0·83,1·03)	0·97 (0·8,1·21)	0·95 (0·83,1·07)

Table D: Summary of results from the spatial dependence analysis describing if 1) a conclusion can be drawn about the relationship between a containment activity and generation of new cases, and ii) the direction of the relationship.

Activity	Conclusion
Fogging	Yes, Decreasing
IRS	Yes, Decreasing
Larviciding	No
Dewatering	No
Tap Fixing	No
Tire Shredding	No

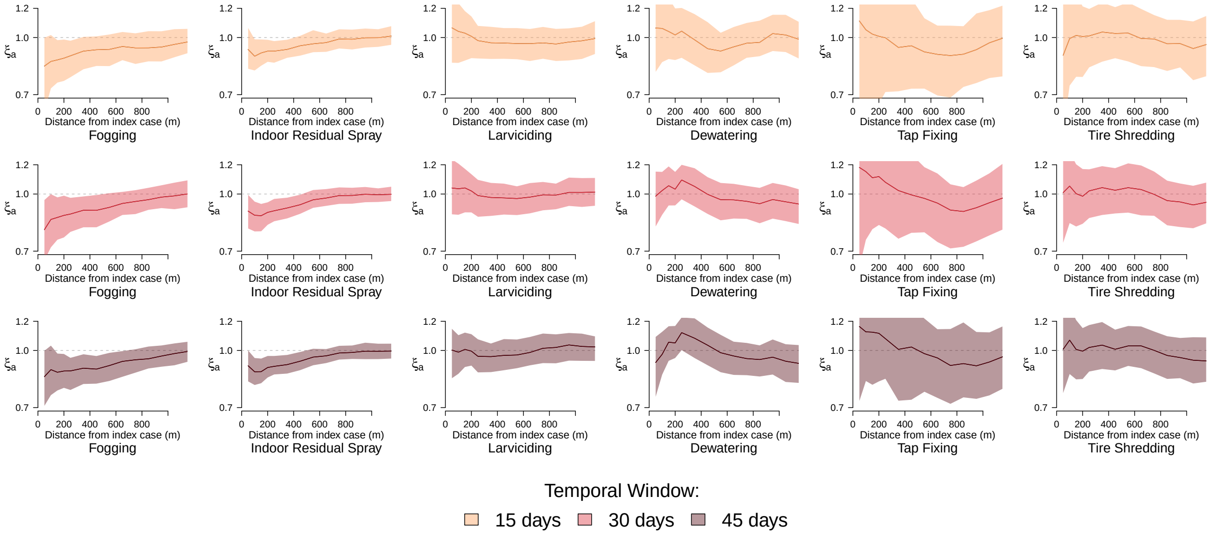

Figure C: Variation in spatial signature ξ_a, verses distance (in meters) from index cases for different time windows (t_2 - t_1) using data from both cities. Values of ξ_a are calculated using time windows (t_2 - t_1) of 15, 30 and 45 days. The value of t_1 is set to 0. Containment and non-containment cases are matched using m= 1000 m. The spatial window of the analysis (d_2 minus d_1) is kept at 500 m when d_2 is greater than 500 m. For d_2 less than 500 m, d_1 is equal to zero. Spatial dependence estimates are plotted at midpoint of the spatial window. Values below 1 show a lower probability of new cases appearing around a case which received containment, compared to a non-containment case. 95% CI is shown as shaded area around estimates.

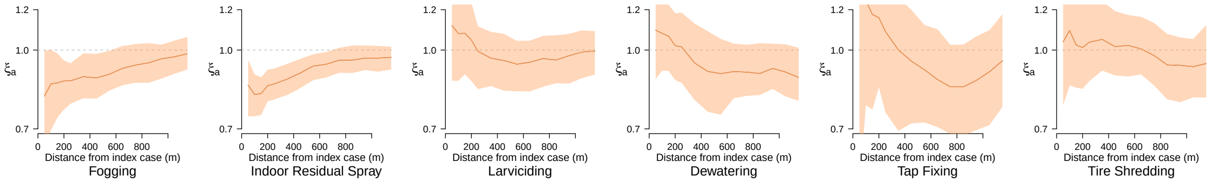

Figure D: Variation in spatial signature ξ_a versus the distance (in meters) from index cases using data from both cities. The values of ξ_a are calculated using control and containment cases which appear in a radius of less than 1000m but greater than 500m radius of each other. The spatial window of the analysis (d_2 minus d_1) is kept at 500 m when d_2 is greater than 500 m. For d_2 less than 500 m, d_1 is equal to zero. Spatial dependence estimates are plotted at midpoint of the spatial window. Values below 1 show a lower probability of new cases appearing around a case which received containment, compared to a control case. The time window $t_2 - t_1$ is set to 30 days. 95% CI is shown as shaded area around estimates.

6. Timeseries (Impact on R_0) analysis - Extended Methods and Results

6.1. Spatial Unit Definition

The city of Lahore being the provincial capital of Punjab has well defined administrative boundaries. Hence for the city of Lahore we use 'Town' administrative units (*N*= 10 towns)– the second smallest administrative unit in the city, as the spatial unit in our study to model localized transmission of dengue. The choice of selecting towns over union council– the smallest administrative unit was made to ensure that each spatial unit had enough reported cases to show variation in the number of cases across time. Additionally, town-level location of each reported dengue patient was recorded in the patient portal, which allowed us to analyze time series of cases in Lahore starting 2012 instead of 2014 when exact geo-location tagging of dengue patients started.

In contrast to the city of Lahore, the administrative boundaries of Rawalpindi are not well defined (as is the case in many cities in low-resource countries). Hence with the help of PITB, the spatial units in Rawalpindi were manually defined accounting for clustering of dengue cases and ensuring approximately consistent area for each unit. To ensure that only areas in the city where cases occurred were included in the study, we only identified spatial units containing cases. This resulted in 14 spatial units for Rawalpindi (Fig. 1 and Table G). The corresponding number of containment activities, provided in Table A, also refer to activities performed within the boundaries of these spatial units.

6.2. Vector Life Cycle and Delayed Effect of Containment Activities

Delays in transmission of dengue between host-vector-host play an integral role in understanding the dynamics of disease spread. Hence, assessments of the effect of containment activities targeted at different stages of the mosquito life cycle need to be adjusted for lagged effects accordingly, based on the stage they are targeted at, when modelling dengue transmission. Despite an *Aedes Aegypti* mosquito getting infected on day 1, a certain number of days are required for the virus to replicate inside the mosquito before it can transmit the virus to a healthy human. Moreover, once getting bitten by an infected mosquito, the virus takes several days to replicate inside a human, before the first symptoms start to appear. Thus, such delays need to be explicitly accounted for in the study for correct estimation of the parameters. There is a $\sim$2 week lag between containment activities targeted at the adult mosquito stage and an expected corresponding decrease in dengue transmission. This is because, once acquired, it takes approximately 4–10 days for the virus to spread inside the vector before it can be transferred to a new host [8]. Moreover, there is an additional delay of 4–7 days before the first symptoms typically appear in the host, once the virus has been introduced in their system [9, 10]. Thus, if adult mosquitoes are eradicated on a given day, infected patients from previous weeks may still appear for 2 weeks after eradication. Containment activities targeted at the larval stage and at source reduction have additional delays of 1-2 weeks, based on the time taken to complete the mosquito life cycle [9]. Hence we lag all containment activities targeted at adult stage of mosquitoes (fogging and IRS), by 1 time step (time step of 2 weeks is used in the study), while activities targeted at larval stage of mosquitoes and source reduction and climate parameters are lagged by 2 time step. Climate parameters were lagged similar to source reduction activities, given that they play an integral role in the growth and development of dengue vector [11, 12].

6.3. Reporting Rate

Unlike other areas and cities where patient data and disease surveillance is performed passively, the cities of Lahore and Rawalpindi are a bed for active surveillance and patient reporting. Instead of patients visiting hospitals on their own accord, special awareness campaigns are carried out to encourage people to visit hospitals even with mild symptoms. The city of Lahore houses over 100 hospitals while the city of Rawalpindi houses over 50 hospitals and hence we find that concerns related to patients not visiting hospitals due to travel cost are negligible. Additionally, free treatments are provided in public hospital. All of these conditions result in high reporting of dengue cases in both cities. To estimate the exact reporting rate in the city, we assume that our dataset consisted of only those patient which were reported from the public hospitals, given the presence of government officials entering the information in the central portal in those

hospitals. Based on a survey published by Pakistan Bureau of Statistics [13], we found that 26.50% individuals living in urban Punjab do not visit private hospitals [14]. Additionally, nearly half of the patients suffering from dengue are asymptomatic and hence are not reported [15]. Thus the reporting rate was assumed to be the product of percentage of symptomatic cases and percentage of individuals who do not visit private facilities. Table F and G show the breakdown of dengue cases before and after correcting for reporting rate across spatial units in Lahore and Rawalpindi.

Sensitivity analysis of the reporting rate was also performed. Figure 4 and 5 (main text) show the variation in the effect of containment activities with changes in reporting rate for models trained for each city. We find that while the magnitude of R_0 for each containment activity changes with changes in reporting rate, the overall direction and statistical significance of the effect of each containment activity is insensitive to the reporting rate.

6.4. Model Parameters, Initial Conditions and Fit

The number of susceptible patients at the start of study was assumed to be the difference between the total population of each spatial unit and the number of confirmed dengue patients, after adjusting for reporting rate, from that spatial unit during the epidemic of 2011 [16]. The population of each spatial unit was inferred and adjusted for over the time period of the study based on previously published work and Worldpop [17, 18]. Birth and death rates were inferred using national statistics from the UNICEF [19]. Data was aggregated at time steps of two weeks. Infected individuals from a previous time step were not counted in following weeks due to the fact that these individuals were admitted in the hospital, quarantined, and removed from the infected population. Recovered individuals were not added in the susceptible population given the fact that dengue patients are immune to future dengue infections from the same strain of virus. The mixing parameter (α_i) was optimized separately for each spatial unit. The number of susceptible, infected and recovered individuals for each spatial unit were calculated as a continuous time series for the entire period of the study.

6.5. Sampling Bias and Modeling Limitations

Sampling bias in the reports considered in our study is minimized due to the fact that each activity was performed by a consistent group of workers from the corresponding specialized government department. Moreover, strict guidelines were provided to the health workers in each department responsible for performing the containment activities as described in the Smartphone Application and Containment Activities section. Also in the implemented methods, our ability to infer the causal impact of each intervention is constrained by the observational nature of the data. Based on correspondence with government officials, placement of the containment activities was strategic, but the amount and timing of the activities could be improved. However, such pragmatic targeting should, if anything, lead us to under-estimate the effectiveness of each intervention [20]. Second, as opposed to using separate models for the transmission of dengue in humans and mosquitoes, we only modeled the transmission of dengue between humans. The consistent effect of rainfall, temperature and population density observed in our study with previous efforts shows that this simplification was reasonable [21]. Finally, we assume that our data on containment activities and cases of dengue are unbiased after correcting for reporting rate – while it is possible that some areas may systematically under- or over-report containment or dengue incidence, we have no way to quantify such measurement error but believe the effect of this to be limited and consistent across all spatial units.

Table E: Relationship between parameters and the R_0 of dengue based on model trained on i) data from Lahore, and ii) from Rawalpindi only. (***) represents p <0.001.

Feature	Type	Application	Relationship with R_0 (data from Lahore)	Relationship with R_0 model (data from Rawalpindi)
Fogging	Adulticide	Targeted	Decreasing(***)	No change( )
IRS	Adulticide	Targeted	Decreasing(***)	Decreasing(***)
Larviciding	Larvicide	Daily Survey	No change( )	No change( )
Fish Seeding	Larvicide	Daily Survey	No change( )	No change( )
Tap Fixing	Source Reduction	Daily Survey	Decreasing(***)	No change( )
Tire Shredding	Source Reduction	Daily Survey	No change( )	Decreasing( )
Dewatering	Source Reduction	Daily Survey	No change( )	No change( )
Temperature	Climate	–	Increasing(***)	Increasing(***)
Rainfall	Climate	–	Increasing(***)	Increasing(***)
Population density	Population	–	Increasing(***)	Increasing(***)

Table F: Population, area (in squared kilometers), reported cases and cases after correction of reporting rate in spatial units in Lahore. Cases represented after correcting reporting rate are average values of 100 iterations.

Spatial Unit	Population (2012)	Area	Reported cases	Cases after Reporting Rate correction
Aziz Bhatti	598138	68	249	1874
Cantonment	855565	97	549	4171
Data Gunj Baksh	1026462	34	370	2864
Gulberg	823116	43	321	2467
Iqbal	817708	516	286	2192
Nishtar	1057829	495	342	2622
Ravi	1676519	46	224	1752
Samnabad	1041605	37	338	2475
Shalamar	561363	24	249	1854
Wagha	694403	437	70	553

Table G: Population, area (in squared kilometers), reported cases and cases after correction of reporting rate in spatial units in Rawalpindi. Cases represented after correcting reporting rate are average values of 100 iterations.

Spatial unit	Population (2014)	Area	Reported cases	Cases after Reporting Rate correction
ISB1	652910	68	198	1484
ISB2	171175	36	40	304
ISB3	40620	18	64	473
ISB4	30339	39	61	481
RK1	31133	10	218	1593
RWP1	364991	55	851	6382
RWP2	526377	38	3032	22623
RWP3	196323	33	1227	9203
RWP4	40693	39	319	2361
RWP5	37265	51	23	155
RWP6	142067	21	794	6047
RWP7	38212	35	636	4749
RWP8	116675	31	146	1083
W1	270334	84	112	772

References

1 Wesolowski A, Qureshi T, Boni MF, Sundsoy PR, Johansson MA, Rasheed SB, et al. Impact of human mobility on the emergence of dengue epidemics in Pakistan. Proc Natl Acad Sci U S A. 2015;112(38):11887–92. doi:10.1073/pnas.1504964112.

2 Rasheed S, Butlin R, Boots M. A review of dengue as an emerging disease in Pakistan. Public Health. 2013;127(1):11–17.

3 Abdur Rehman N, Kalyanaraman S, Ahmad T, Pervaiz F, Saif U, Subramanian L. Fine-grained dengue forecasting using telephone triage services. Sci Adv. 2016;2(7):e1501215. doi:10.1126/sciadv.1501215.

4 Organization WH. Regional Office for South East Asia: Comprehensive Guidelines for Prevention and Control of Dengue and Dengue Hemorrhagic Fever: Revised and expanded edition. New Delhi, India. 2011;14:16.

5 Luz PM, Vanni T, Medlock J, Paltiel AD, Galvani AP. Dengue vector control strategies in an urban setting: an economic modelling assessment. Lancet. 2011;377(9778):1673–80. doi:10.1016/S0140-6736(11)60246-8.

6 WHO SPECIFICATIONS AND EVALUATIONS FOR PUBLIC HEALTH PESTICIDES;. http://www.who.int/whopes/quality/Temephos_eval_only_June_2011.pdf.

7 Deltamethrin | C22H19Br2NO3 - PubChem;. https://pubchem.ncbi.nlm.nih.gov/compound/deltamethrin.

8 Dengue and severe dengue;. http://www.who.int/en/news-room/fact-sheets/detail/dengue-and-severe-dengue.

9 Christophers S. Aedes aegypti (L.) the yellow fever mosquito: its life history, bionomics and structure. Rickard. 1960;.

10 Epidemiology | Dengue | CDC;. https://www.cdc.gov/dengue/epidemiology/index.html.

11 Bueno-Mari R, Jimenez-Peydro R. Global change and human vulnerability to vector-borne diseases. Front Physiol. 2013;4:158. doi:10.3389/fphys.2013.00158.

12 Xu L, Stige LC, Chan KS, Zhou J, Yang J, Sang S, et al. Climate variation drives dengue dynamics. Proceedings of the National Academy of Sciences. 2017;114(1):113–118.

13 PAKISTAN SOCIAL AND LIVING STANDARDS MEASUREMENT SURVEY (2004-05);. http://www.pbs.gov.pk/sites/default/files/social_statistics/publications/pslm2004-05/pslms%202004-05.pdf.

14 Akbari AH, Rankaduwa W, Kiani AK. Demand for public health care in Pakistan. The Pakistan Development Review. 2009; p. 141–153.

15 Bhatt S, Gething PW, Brady OJ, Messina JP, Farlow AW, Moyes CL, et al. The global distribution and burden of dengue. Nature. 2013;496(7446):504–7. doi:10.1038/nature12060.

16 Ali Z, Zahra G, Khan B, Ali H, Bibi F, Khan A. Prevalence of dengue fever during 2011-2012 in Punjab. The Journal of Animal and Plant Sciences. 2015;25:348–354.

17 Shirazi SA. Spatial analysis of NDVI and density of population: a case study of lahore-pakistan. Science International. 2012;24(3).

18 Worldpop;. http://www.worldpop.org.uk/.

19 Statistics | Pakistan | UNICEF;. https://www.unicef.org/infobycountry/pakistan_pakistan_statistics.html.

20 Reiner J R C, Achee N, Barrera R, Burkot TR, Chadee DD, Devine GJ, et al. Quantifying the Epidemiological Impact of Vector Control on Dengue. PLoS Negl Trop Dis. 2016;10(5):e0004588. doi:10.1371/journal.pntd.0004588.

21 Kraemer MU, Perkins TA, Cummings DA, Zakar R, Hay SI, Smith DL, et al. Big city, small world: density, contact rates, and transmission of dengue across Pakistan. J R Soc Interface. 2015;12(111):20150468. doi:10.1098/rsif.2015.0468.
